# Multiscale stochastic modeling for calcium dynamics in cardiac electrophysiology: assessing whole-cell model reliability under phosphorylation and LCC downregulation

**DOI:** 10.3389/fnetp.2026.1727426

**Published:** 2026-04-13

**Authors:** Gustavo Montes Novaes, Rodrigo Weber Dos Santos, Sergio Alonso, Enrique Alvarez-Lacalle

**Affiliations:** 1 Department of Computing, Federal Center for Technological Education of Minas Gerais, Leopoldina, Brazil; 2 Graduate Program in Computational Modeling, Federal University of Juiz de Fora, Juiz de Fora, Brazil; 3 Department of Physics, Universitat Politècnica de Catalunya-BarcelonaTech, Barcelona, Spain; 4 Institute for Research and Innovation in Health, Universitat Politècnica de Catalunya-BarcelonaTech, Barcelona, Spain

**Keywords:** calcium release unit, computational modeling, L-type calcium channel, Markov chain, myocytes, ryanodine receptors, network physiology

## Abstract

Intracellular calcium (Ca^2+^) dynamics drives contractile function in cardiac myocytes. In particular, L-type Calcium Channels (LCCs) and Ryanodine Receptors (RyRs) are organized in microdomains, where LCCs trigger substantial Ca^2+^ release from the Sarcoplasmic Reticulum (SR) via RyRs. Different microdomains can be coupled at different length scales by calcium diffusion or common external activation. We present a Scalable Aggregate Calcium Release Unit (SA-CaRU) model for human ventricular myocytes that integrates a recently developed Markov Chain (MC)-based description of LCCs, replacing classical Hodgkin–Huxley gates. Our approach is based on previously published MC-based frameworks for the human heart, enabling stochastic gating and reproducing evoked local Ca^2+^ release statistics across different effective levels of microdomain aggregation. Our single-SA-CaRU system captures, within a unified framework, key features of microscale and macroscale Ca^2+^ cycling and allows, for the first time, systematic exploration of variability in SR Ca^2+^ release as a function of effective microdomain size and coupling. Simulations with increasing numbers of channels reveal that the transition from stochastic to deterministic-like Ca^2+^ behavior is typically sharp at a specific cluster size. Under normal (healthy) conditions, this occurs at $O\left(10^{2}\right)$ LCCs (with mild sensitivity to the RyR:LCC scaling). However, under high phosphorylation or LCC upregulation, stochasticity persists and convergence to deterministic-like behavior is absent or markedly delayed even for total LCC numbers as large as 20,000. In these conditions, whole-cell deterministic models become doubtful, since their behavior can be qualitatively different from that arising from any plausibly mediated coordination of subcellular calcium release units.

## 1 Introduction

The heart is a mechanical pump and contraction is regulated by an electrical signal that travels from cell to cell, known as the action potential (Malmivuo and Plonsey, 1995). The disruption in the synchronization of these electrical impulses can lead to various arrhythmias, including atrial flutter, atrial fibrillation, ventricular fibrillation, and ventricular tachycardia (Zipes et al., 2017). Some of such arrhythmias are fatal, making cardiovascular disease the leading cause of death in developed countries (Mendis et al., 2011; Timmis et al., 2020).

Clinical studies have shown that the trigger for this arrhythmia can be localized excitation, which can lead to premature ectopic beat. This abnormal excitation can appear due to micro-reentries (Oliveira et al., 2018; Alonso et al., 2016) or due to the interaction of abnormal calcium cycling and sodium currents in cardiac cells (Clusin, 2003; Wehrens et al., 2005; Xie et al., 2015). Such suggestions have been further studied using mathematical models of cardiac tissue (Alonso et al., 2025; Niederer et al., 2019), which basically describe the electrophysiological properties of cardiac cells and, in particular, the dynamics of the action potential. Such models have suggested that malfunctions in calcium cycling can trigger ectopic beat action at the single-cell level, both as early afterdepolarizations (EADs) and as delayed afterdepolarizations (DADs) (Colman et al., 2022; Chen et al., 2009).

Cardiac contraction is based on highly regulated intracellular Ca^2+^ cycling. At the onset of each heartbeat, upon cell depolarization, L-type Calcium Channels (LCCs) open and allow moderate Ca^2+^ influx through the membrane, triggering the opening of RyR2 in clusters in front of the LCC initiating a dramatic release of Ca^2+^ from the sarcoplasmic reticulum (SR) in a process known as Calcium-Induced Calcium Release (CICR). The calcium released must be later reuptaken into the SR via the SR Ca pump (SERCA) and outside the cell, mainly via the sodium-calcium exchanger (NCX). The joint structure of the RYR2 receptor cluster and the LCC in front of them, while each is in a different membrane, is tightly packed with a small dyadic volume between them. They represent a coherent calcium release unit (CaRU) at the core of heart contraction. These microdomains are distributed along the Z-planes of the cardiomyocyte and associated with invagination of the cell membrane, t-tubules, with a characteristic distance between them of 300–500 nm within the plane and a wide distribution of sizes. Between the Z-planes, roughly 2 μ m apart, some RyR2 clusters without associated LCC are also present, but they do not constitute the core of the normal calcium transient and are very sparse.

Although the core of cardiac ${\text{Ca}}^{2+}$ cycling originates in discrete microdomains, modeling efforts have often operated at the whole-cell scale using deterministic models that treat the whole cell as single set of typically five compartments, such as the ones developed by Pandit et al. and ten Tusscher and Panfilov (Pandit et al., 2001; Ten Tusscher and Panfilov, 2006), to simulate the global behavior of action potentials and calcium transients. Necessarily, they treat ionic currents through population-level averages using Hodgkin-Huxley-type formulations, and the stochastic opening and closing of individual ion channels and the resulting local fluctuations are not considered or studied. Subcellular behavior such as the rate of stochastic local releases (sparks) during diastole or the possible presence of diastolic calcium waves (Sato et al., 2013; Colman et al., 2022), where the probabilistic interaction between a small number of L-type ${\text{Ca}}^{2+}$ channels (LCCs) and neighboring ryanodine receptors (RyRs) within the dyadic cleft plays a crucial role, is not accounted for. Any relevance they might have to the global average dynamics of a normal heartbeat is ignored. This means that other possible sources of pro-arrhythmic behavior may be outside of the scope of these models (Colman et al., 2022).

To address these local phenomena, subcellular models have emerged that treat the calcium release unit (CaRU) as the fundamental computational element, incorporating thousands of discrete CaRUs where a small number of LCCs and RyRs are present (Marchena and Echebarria, 2018). These units are coupled directly by calcium through diffusion and indirectly via a common membrane voltage. Their stochastic dynamics require the use of either Monte Carlo techniques or Markov Chain (MC) state diagrams. Such approaches can be used to reproduce critical features such as ${\text{Ca}}^{2+}$ sparks, brief localized calcium releases from the SR, as well as spark-mediated ${\text{Ca}}^{2+}$ waves that can propagate across the cell.

Bridging the gap between these two modeling paradigms—whole-cell deterministic and subcellular stochastic-is a major challenge. The behavior of individual CaRUs does not scale trivially to the whole cell level, as spatial organization, local calcium diffusion, and the nonlinearities of CICR introduce emergent behaviors not predictable from averaged dynamics. The computational burden of simulating thousands of stochastically coupled release units with spatial resolution is significant, making the simulation of whole organ behavior using subcellular models numerically impossible. Proper whole-cell organ models require an understanding of the relationship between the two approaches. More specifically, understanding when the average behavior of calcium release units can be properly translated into whole-cell dynamics is crucial. When local and global behaviors diverge under specific conditions, whole-cell deterministic models must be carefully adapted for studies at the whole-heart scale.

In this manuscript, to address the stochastic behavior of the calcium-induced calcium release (CICR) process, we define a Scalable Aggregate Calcium Release Unit (SA-CaRU) in which both LCC and RyR channels are modeled as MCs. We simulate the dynamics of a single cluster and systematically analyze the variability of Ca^2+^ release as a function of the number of channels within the SA-CaRU (i.e., cluster size), systematically varying the number of LCC and RyR channels from a single LCC channel and 5 RyRs to thousands of channels within the same SA-CaRU. An SA-CaRU with a low number of LCC reproduces the behavior of a real cluster of RyR, a real Calcium Release Unit. For slightly larger values of LCC, it captures the behavior of macrodomains coupled by diffusion, and for a large number of LCC, it reproduces a mean-field analysis with unrealistically high diffusion, allowing us to check whether it captures average whole-cell behavior. We want to stress that the SA-CaRU does not represent a single enlarged microdomain when the number of channels is very large. Rather, it is a mean-field statistical description of many independent CRUs, analogous to population-density approaches. No assumption of instantaneous or perfect local coupling is made; CRUs interact only through the global cytosolic Ca concentration. This formulation preserves known spatial constraints of dyadic microdomains while capturing the collective stochastic behavior of CICR, allowing the study of the system at different scales.

We consider both the original parameters and two sets of key parameters that emulate the common adjustments made in whole-cell models. The first set mimics conditions observed in patients with marked downregulation of LCCs, while the second represents increased pumping activity mediated by phosphorylation. The robustness of the relationship between deterministic whole-cell models and the subcellular coupling among a few microdomains under these conditions provides important insights into the adequacy and limitations of whole-cell approaches.

Our results reveal a sharp behavioral transition around 100 L-type calcium channels (LCCs) in healthy cardiomyocytes, marking a critical threshold where calcium release shifts from a stochastic to a more deterministic regime. Below this threshold, release events display high variability, primarily driven by random fluctuations in the opening and closing of individual channels. In contrast, under LCC downregulation, and especially under conditions of strong phosphorylation, the distinction between deterministic and stochastic regimes persists even when more than 10,000 channels are involved. In these cases, deterministic whole-cell models fail to reproduce physiologically realistic behavior. Instead, the subcellular system exhibits intrinsically stochastic dynamics characterized by a bimodal distribution, alternating between large and very large calcium release events that can only be captured by an explicitly stochastic subcellular model.

## 2 Methods

We begin with a baseline deterministic cell model and introduce stochastic Markov Chain representations for L-type Calcium Channels (LCCs) and Ryanodine Receptors (RyRs). This approach enables us to adapt subcellular calcium compartments and fluxes to exhibit both deterministic and probabilistic behaviors depending on the channel densities. We can define in this way a Scalable Aggregate calcium Release Unit (SA-CaRU) that, depending on he number of LCC channels and RyRs receptors, will move from a purely stochastic behavior at a very low number of channels towards a full deterministic model when the number of channels goes to infinity. We proceed to describe the details of this general idea.

### 2.1 Electrophysiological model

We take as our original model, which we then modify, the ten Tuscher model for the human ventricle cell (Ten Tusscher and Panfilov, 2006). We replace the original Hodgkin-Huxley (HH)-type gating formulations with a novel representation based on Markov Chain (MC), which produces an equivalent response to experimental conditions (Novaes et al., 2022).

**FIGURE 1:**
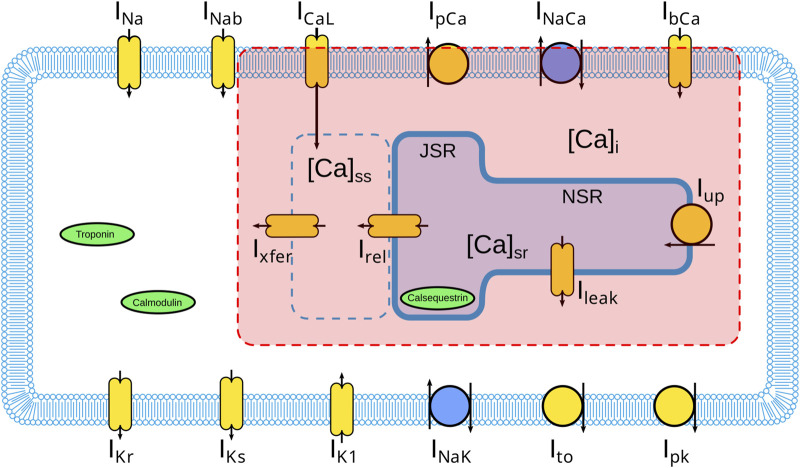
Illustration of a schematic representation of the novel MC-based Model highlighting, in red, the structures that compose a single Scalable Aggregate Calcium Release Unit (SA-CaRU) which would reproduce both a real cluster of LCC-RyR for low $N_{\mathrm{LCC}}$ and would provide a mean field approach for a number of $N_{\mathrm{LCC}}$ similar to the ones present in a whole cell. A more detailed explanation of the proper interpretation of the SA-CaRU is given in the main text.

More specifically, in the base model used in this work, since cardiac cells are separated from the neighboring extracellular space by a phospholipid bilayer membrane, the cell is considered a capacitor that stores electric charge. The total electrical current through the cell membrane is equal to the change in the charge of the membrane, giving rise to the standard single-cell models characterized by the change in the membrane potential:

$$C_{m}\frac{dV}{dt}=-\left(I_{\mathrm{ion}}+I_{st}\right);$$ (1)

where $V=V_{i}-V_{e}$ in Equation 1 is the electrical potential of the membrane, $C_{m}$ is the capacitance of the membrane and the term $I_{st}$ consists of an external stimulus or pacing. In our model, we adopt a popular formulation of the total ionic current (Ten Tusscher and Panfilov, 2006), which includes contributions from sodium, potassium, and calcium currents. Mathematically, it is defined as:

$$I_{\mathrm{ion}}=I_{Na}+I_{K1}+I_{to}+I_{Kr}+I_{Ks}+I_{\mathrm{CaL}}+I_{\mathrm{NaCa}}+I_{\mathrm{NaK}}+I_{\mathrm{pCa}}+I_{pK}+I_{\mathrm{bCa}}+I_{\mathrm{bNa}},$$ (2)

where the terms in Equation 2 represent ionic fluxes through voltage-gated channels, pumps, and exchangers, see Figure 1. To provide a full cell model, sodium, potassium, and calcium cycling have to be included to track the concentration of ions inside the cell. So, in order to complete the single-cell description, intracellular ionic concentrations are dynamically computed, with particular emphasis on the calcium-handling system, which is central to the excitation–contraction coupling mechanism and, therefore, to this study.

We change the $I_{\mathrm{CaL}}$ and the calcium cycling of the original ten Tusscher model (Ten Tusscher and Panfilov, 2006) in terms of a Scalable Aggregate Calcium Release Unit (SA-CaRU). More specifically, we modify the L-type calcium current $\left(I_{\mathrm{CaL}}\right)$ and the calcium cycling subsystem of the original ten Tusscher model to incorporate a Markov Chain–based representation leading to a stochastic framework (Novaes et al., 2022) which allows us to describe the probabilistic opening and closing of L-type Calcium Channels (LCCs) and their interaction with Ryanodine Receptors (RyRs) within a Scalable Aggregate Calcium Release Unit (SA-CaRU). The SA-CaRU provides a unified structure that encompasses the possibility to simulate channel-level stochasticity and whole-cell-level calcium signaling, allowing us to investigate the gap between local microdomain behavior and global calcium cycling. The general structure of the model is provided in Figure 1 and we proceed to provide details.

### 2.2 Scalable aggregate CRU setup

Our focus is to study an Scalable Aggregate Calcium Release Unit (SA-CaRU), composed of a cluster of LCCs and RyRs governed by a Markov Chain process within a dyadic domain. In our framework, the SA-CaRU does not represent only a single anatomical microdomain; rather, it serves as a flexible abstraction that can range from an individual subcellular Ca^2+^ release unit to a coordinated cluster of microdomains, and even up to a cell-wide release structure. This allows us to investigate calcium dynamics on different spatial and organizational scales using a unified modeling approach.

Let $N_{\mathrm{LCC}}$ and $N_{\mathrm{RyR}}$ denote the number of L-type calcium channels and ryanodine receptors in the cluster, respectively. A typical single microdomain configuration might use $\left(N_{\mathrm{LCC}},N_{\mathrm{RyR}}\right)=\left(4,20\right)$ (Bers and Stiffel, 1993; Qu et al., 2022). However, changing and increasing the number of LCC and RyR, we can explore how stronger coordination between microdomains works. Our model offers flexibility in fully exploring up to very large values of $N_{\mathrm{LCC}}$ and $N_{\mathrm{RyR}}$ , which should reproduce the actual models of single-cell behavior.

In this single unit, local calcium concentrations, such as ${\left[Ca\right]}_{ss}$ (calcium in the subsarcolemma), ${\left[Ca\right]}_{SR}$ (calcium in the Sarcoplasmic Reticulum), and ${\left[Ca\right]}_{i}$ (cytosolic calcium), are determined by fluxes resulting from $I_{\mathrm{CaL}}$ , $I_{\mathrm{rel}}$ , and $I_{up}$ , similar to standard deterministic modeling approaches for the total cell. The governing equations, see Equations 3–9, for these variables read:

$$\frac{d{\left[Ca\right]}_{i}}{dt}=-\frac{I_{\mathrm{bCa}}+I_{\mathrm{pCa}}-2I_{\mathrm{NaCa}}}{2V_{c}F}+\frac{V_{sr}}{V_{c}}\left(I_{\mathrm{leak}}-I_{up}\right)+I_{\mathrm{xfer}},$$ (3)

$$\left\{\begin{array}{c} {{\left[Ca\right]}_{i}}_{\mathrm{buff}}=\frac{{\left[Ca\right]}_{i}\times Buf_{i}}{{\left[Ca\right]}_{i}+K_{\mathrm{bufi}}},\\ {\left[Ca\right]}_{i}={{\left[Ca\right]}_{i}}_{\mathrm{free}}+{{\left[Ca\right]}_{i}}_{\mathrm{buff}}, \end{array}\right.$$ (4)

$$\frac{d{\left[Ca\right]}_{SR}}{dt}=\left(I_{up}-I_{\mathrm{leak}}-I_{\mathrm{rel}}\right),$$ (5)

$$\left\{\begin{array}{c} {{\left[Ca\right]}_{SR}}_{\mathrm{buff}}=\frac{{\left[Ca\right]}_{SR}\times Buf_{sr}}{{\left[Ca\right]}_{SR}+K_{\mathrm{bufsr}}},\\ {\left[Ca\right]}_{SR}={{\left[Ca\right]}_{SR}}_{\mathrm{free}}+{{\left[Ca\right]}_{SR}}_{\mathrm{buff}}, \end{array}\right.$$ (6)

$$\frac{d{\left[Ca\right]}_{ss}}{dt}=-\frac{I_{\mathrm{CaL}}}{2V_{ss}F}+\frac{V_{sr}}{V_{ss}}I_{\mathrm{rel}}-\frac{V_{c}}{V_{ss}}I_{\mathrm{xfer}},$$ (7)

$$\left\{\begin{array}{c} {{\left[Ca\right]}_{ss}}_{\mathrm{buff}}=\frac{{\left[Ca\right]}_{ss}\times Buf_{ss}}{{\left[Ca\right]}_{ss}+K_{\mathrm{bufss}}},\\ {\left[Ca\right]}_{ss}={{\left[Ca\right]}_{ss}}_{\mathrm{free}}+{{\left[Ca\right]}_{ss}}_{\mathrm{buff}},\text{ and} \end{array}\right.$$ (8)

$$I_{\mathrm{xfer}}=V_{\mathrm{xfer}}\left({{\left[Ca\right]}_{ss}}_{\mathrm{free}}-{{\left[Ca\right]}_{i}}_{\mathrm{free}}\right).$$ (9)

Fluxes related to NCX activity $I_{\mathrm{NaCa}}$ , calcium pump $I_{\mathrm{pCa}}$ , calcium background current into the cell $I_{\mathrm{bCa}}$ , and the leak of calcium from the SR into the cytosol $I_{\mathrm{leak}}$ follow the same formulation as in the original ten Tusscher model. They read:

$$I_{\mathrm{NaCa}}=k_{\mathrm{NaCa}}\frac{e^{\gamma VF/RT}Na_{i}^{3}{\left[Ca\right]}_{o}-e^{\left(\gamma -1\right)VF/RT}Na_{o}^{3}{{\left[Ca\right]}_{i}}_{\mathrm{free}}\alpha }{\left(K_{\mathrm{mNai}}^{3}+Na_{o}^{3}\right)\left(K_{\mathrm{mCa}}+{\left[Ca\right]}_{o}\right)\left(1+k_{\mathrm{sat}}e^{\left(\gamma -1\right)VF/RT}\right)},$$ (10)

$$I_{\mathrm{pCa}}=G_{\mathrm{pCa}}\frac{{{\left[Ca\right]}_{i}}_{\mathrm{free}}}{K_{\mathrm{pCa}}{{\left[Ca\right]}_{i}}_{\mathrm{free}}},$$ (11)

$$I_{\mathrm{bCa}}=G_{\mathrm{bCa}}\left(V-E_{Ca}\right),\text{ and}$$ (12)

$$I_{\mathrm{leak}}=V_{\mathrm{leak}}\left({{\left[Ca\right]}_{SR}}_{\mathrm{free}}-{{\left[Ca\right]}_{i}}_{\mathrm{free}}\right);$$ (13)

The different parameters in Equations 10–13 also come from the ten Tusscher model. For more details, see the original model (Ten Tusscher and Panfilov, 2006). We proceed now to describe the other key currents.

### 2.3 L-type calcium channel

**FIGURE 2:**
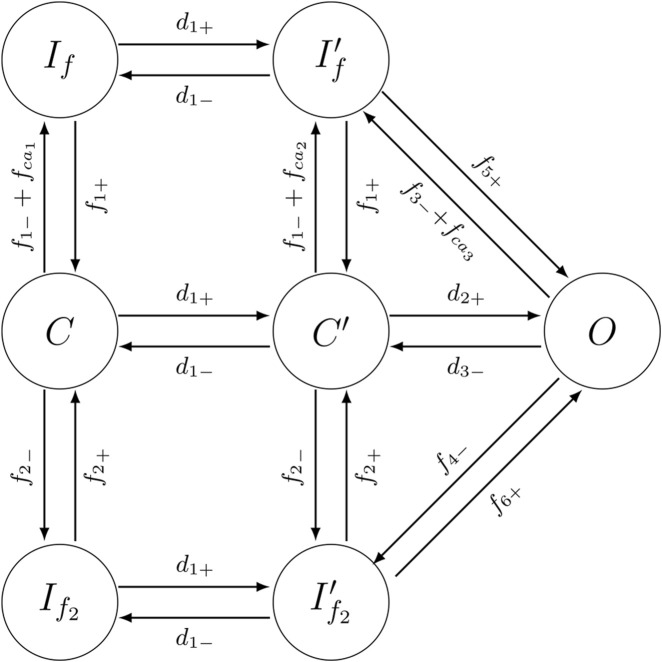
The proposed Markov Chain formulation for the L-type calcium channel $\left(I_{\mathrm{CaL}}\right)$ , adapted from (Novaes et al., 2022). This scheme integrates Hodgkin-Huxley–inspired rate functions with the state topology originally proposed in (Mahajan et al., 2008), replacing the gate-based formulation in the ten Tusscher model (Ten Tusscher and Panfilov, 2006). Transitions associated with activation and inactivation are incorporated into the Markov Chain framework through forward and backward rates between closed, open, and inactivated states ( $f_{1}$ to $f_{6}$ and $d_{1}$ to $d_{3}$ ), whose functional forms embed voltage-dependent modulation. Additional transitions account for calcium-dependent regulation of channel availability through calcium-dependent rate terms ( $f_{{ca}_{1}}$ to $f_{{ca}_{3}}$ ). A detailed description of all transition rates and its dependence on calcium and/or voltage are provided in the Supplementary Material.

The original ten Tusscher model uses a configuration of this channel based on HH-gated variables, which does not have an equivalent stochastic counterpart. Therefore, we define the L-type current as in (Novaes et al., 2022), where each channel can be in seven different configurations represented by a seven-state Markov Chain, see Figure 2, and was designed to represent the dynamics of the original ten Tusscher model. Therefore, the seven-state L-type Ca channel Markov model employed in this study was previously introduced and fully validated (Novaes et al., 2022). That work demonstrated that the model reproduces canonical I–V relationships and ion channel dynamics. When embedded in the ten Tusscher ventricular framework, it also preserved action potential morphology, Ca transient amplitude, restitution behavior, and Ca cycling stability across pacing rates. Because the present manuscript focuses specifically on the stochastic-to-deterministic transition, we use the same validated formulation without modification. There are also different types of models of the cardiac L-type calcium current (Agrawal et al., 2023).

The channel transitions between states in a stochastic manner. The key state responsible for generating the current is the open state $O_{LCC}$ , as depicted in Figure 2. When the channel occupies this state, the resulting current is given by:

$$I_{\mathrm{CaL}}=\frac{O_{\mathrm{LCC}}}{N_{\mathrm{LCC}}}G_{\mathrm{CaL}}\frac{\Delta VF^{2}}{RT}\left(\frac{{\left[Ca\right]}_{ss}e^{\frac{2\Delta VF}{RT}}-4{\left[Ca^{2+}\right]}_{o}}{e^{\frac{2\Delta VF}{RT}}-1}\right),$$ (14)

where ΔV=V−15 in Equation 14. When the channel is in any other state different from the open state, it does not generate current. When in the open state current is produced.

We must emphasize that the transitions between the LCC states are stochastic and that, in the limit of a very large number of channels in the SA-CaRU we revisit the deterministic whole-cell model. It will be important to track the fluctuations when we consider a low number of channels. For this, we use Tau leaping to efficiently model systems with discrete events. This is achieved by exploiting the binomial distribution to represent the number of successful transitions of states (Tian and Burrage, 2004). For each transition with rate k , the number of channels that changed state during a time step Δt was drawn from a binomial distribution with probability of success $p=1-e^{-k\Delta t}$ . This approach guarantees non-negative populations and avoids excessive-leap instabilities associated with classical tau-leaping. All simulations used Δt=τ=0.001 ms , which satisfies kΔt≪1 over the full range of channel rates. Using this approach, the model captures the stochastic dynamics of the system while significantly reducing the computation time compared to simulating each event individually.

Transitions implement the voltage-dependent opening, calcium-dependent inactivation, and closed or inactivated states discussed in other models [as in rabbit (Mahajan et al., 2008)]. This formulation has been shown to adequately reproduce the deterministic evolution of a healthy human ventricular cardiomyocyte (Novaes et al., 2022).

Section 2.1 in the Supplementary Material provides a description of the different rates that define the stochastic process in the L-Type calcium channel. Most of the parameters were taken obtained using a genetic algorithm to search for a proper reproduction of the whole-cell model in the human ventricle (Novaes et al., 2019).

The basic structure of these parameters was designed to provide a more biophysically faithful representation of how a modest trigger current can initiate a large-scale release of calcium from the SR while keeping the basic properties already adequately described in whole-cell models.

Finally, we will compare the stochastic behavior with the exact deterministic behavior coming from this set-up. In this case, each of the Markov Chains becomes an occupation number that tracks a set of differential equations. These differential equations are reproduced numerically.

### 2.4 Calcium release from the SR into the cytosol. The RyR2 model

**FIGURE 3:**
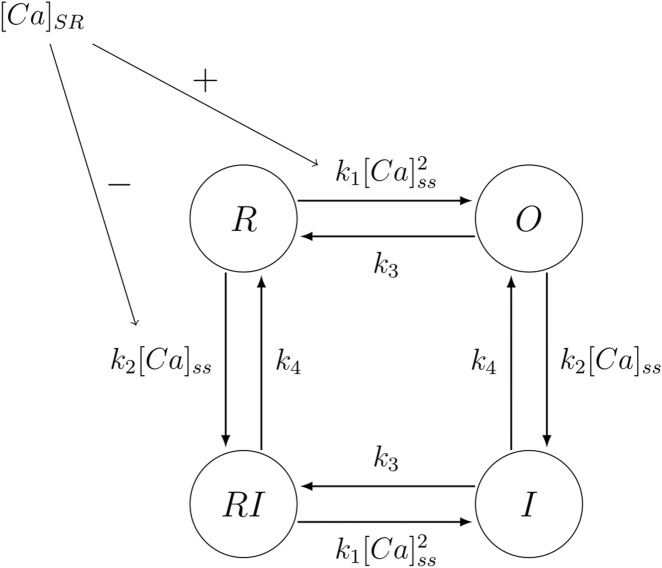
Four-state Markov Chain used to represent the states that the RyR channels can assume. The state I represents an Inactivated closed state, the state R represents a Resting closed state, the state RI represents a Resting Inactivated closed state, and the state O represents the Open conducting state.

Calcium release flux through RyR channels is activated when the receptor is in the open state and is modeled proportional to the calcium concentration gradient. The flux is given by:

$$I_{\mathrm{rel}}=\frac{O_{\mathrm{RyR}}}{N_{\mathrm{RyR}}}V_{\mathrm{rel}}\left({\left[Ca\right]}_{SR}-{\left[Ca\right]}_{ss}\right).$$ (15)

where the variable $O_{RyR}$ in Equation 15 indicates whether the RyR is in the open state. We used a RyR model with four different states as shown in Figure 3, one closed, one open, and two inactivated. This configuration of the calcium release flux model through RyR was already used in the original model (Ten Tusscher and Panfilov, 2006).

The four-state model of RyR has cytosolic calcium regulation corresponding to the calcium-induced calcium-release mechanism (CICR) and inactivation is also directly mediated by cytosolic calcium, mimicking the regulation of the Ca-calmodulin complex (Wei et al., 2021). Furthermore, in this RyR model, both the transitions from the resting state to the open (O) state or the resting-inactivated (RI) state are also regulated by the calcium concentration of the sarcoplasmic reticulum (SR), capturing luminal control of channel gating (Shannon et al., 2004).

Table 1 provides the list of parameters needed to perform the stochastic simulation of the states of the Markov chain. Again, to simulate the number of RyR in the open state, we stochastically computed the number of multiple events that occur within a specified time interval (Δt) using a Tau leaping approach as in the previous section. We will also need to compare with the full set of equations for calcium cycling that is provided in the Appendix.

**TABLE 1:** Formulations of the transition rates for the RyR Markov Chain model. Rates $k_{1}$ and $k_{2}$ are modulated by sarcoplasmic reticulum (SR) calcium concentration and γ respectively, which enable model modulation under different physiological or pathological conditions. For ϕ=1 and γ=1 we recover the original parameter values.

$I_{\mathrm{rel}}$ rate equations		
Parameter	Expression/Value	Units
$k_{1}$	$\frac{k_{1}^{\prime }}{k_{\text{casr}}}$	${\text{mM}}^{-2}\cdot {\text{ms}}^{-1}$
$k_{2}$	$k_{2}^{\prime }\cdot k_{\text{casr}}$	${\text{mM}}^{-1}\cdot {\text{ms}}^{-1}$
$k_{3}$	0.060	${\text{ms}}^{-1}$
$k_{4}$	0.000015	${\text{ms}}^{-1}$
$k_{\text{casr}}$	${\text{max}}_{\text{sr}}-\frac{{\text{max}}_{\text{sr}}-{\text{min}}_{\text{sr}}}{1+{\left(\frac{EC}{{\left[Ca\right]}_{\text{SR}}}\right)}^{2}}$	Dimensionless
$k_{1}^{\prime }$	0.15	${\text{mM}}^{-2}\cdot {\text{ms}}^{-1}$
$k_{2}^{\prime }$	0.045	${\text{mM}}^{-1}\cdot {\text{ms}}^{-1}$
${\text{max}}_{\text{sr}}$	2.5	Dimensionless
${\text{min}}_{\text{sr}}$	1.0	Dimensionless
EC	1.5	mM

### 2.5 Modeling of calcium cycling alterations

We implemented two modifications in the parameters of the SA-CaRU model by targeting two sets of key parameters which aim to reproduce two characteristic features of, first, calcium regulation via LLC regulation due to genetic diseases and, second, calcium regulation via specific phosphorylation.

We use a single parameter indicator ϕ to indicate changes in phosphorylation and another single parameter η for up or downregulation of LCC channels. We describe how we propose to implement these changes.

#### 2.5.1 LCC regulation

The lower expression of LCCs, corresponding to downregulation, decreases the inward calcium current, which is crucial to trigger calcium release from the sarcoplasmic reticulum (Shaw and Colecraft, 2013). The decrease in calcium influx has important consequences for cardiac cell excitability and results in altered cell contractility (Triggle, 1998) and disturbed electrical activity. Finally, such downregulation alters the configuration of the action potential, influencing the overall cardiac rhythm (Benitah et al., 2010).

Increased LCC function, corresponding to upregulation, induces an elevated inward calcium current in cardiac cells, which enhances calcium-triggered calcium release from the sarcoplasmic reticulum. This increase in channel open probability can increase the probability of atrial fibrillation (Klein et al., 2003). The increase in cytosolic calcium transients can improve the strength of cardiac muscle contraction (Zhao et al., 2009). Finally, high LCC activity prolongs the plateau phase of the cardiac action potential and can increase the probability of arrhythmia (Satin and Schroder, 2009).

We model the regulation of the LCC channels by the modification of the inactivation rates, see Figure 2:

$$f_{-}=\eta \left(1-f_{\inf}\right)/\tau_{f}.$$ (16)

$${f_{2}}_{-}=\eta \left(1-{f_{2}}_{\inf}\right)/\tau_{f_{2}}$$ (17)

where we have included the extra parameter η . We have simulated the decrease η=0.5 and the increase η=1.5 of the inactivation rates, i.e., up and downregulation of the LCC channels, respectively, in Equations 16, 17. The modification of these two rates is also equivalent to the increase and decrease of the rate $d_{+}$ , see Figure 2, as implemented in Sato et al. (2018) to model the cooperative gating of L-type ${\text{Ca}}^{2+}$ channels (Sato et al., 2018).

#### 2.5.2 Effect of phosporylation

Protein kinase A (PKA) regulates the cardiac excitation–contraction coupling because it can be activated by cAMP and phosphorylate critical proteins in the channels LCC, RyR, and, phosphorylates also Phospholamban (PLB), aftecting SERCA. Therefore, PKA has a important influence on calcium dynamics and the contractility of the myocytes. Alterations in RyR, LCC and SERCA phosphorylations play a critical role in various cardiac diseases, including heart failure.

The effects of PKA are threefold. First, PKA prolongs the opening time of LCC and increases calcium influx into the cell (Ahern et al., 2019; Ahern et al., 2021). This modification of LCC activity increases SR ${\text{Ca}}^{2+}$ release, promotes ${\text{Ca}}^{2+}$ -dependent remodeling and prometes spontaneous SR ${\text{Ca}}^{2+}$ -release events, To model such increase, we multiplied the transition rate from the $C^{\prime }$ to O state - $t_{C^{\prime }\to O}$ - of the LCC MC (see Figure 2) by a factor ϕ similar to some downregulation approaches (Sato et al., 2018). So, the updated version of the transition rate $t_{C^{\prime }\to O}$ reads

$$t_{C^{\prime }\to O}=\phi x_{7}d_{+}.$$ (18)

Second, PKA also affect the behavior of the RyR2 channels, since the RyR2 has some important phosphorylation sites (Dobrev and Wehrens, 2014) that change the stability of the different states upon the binding of the phosphate group. The key effect of this phosphorylation is to increase the opening rate probability of the RyR2 increasing the probability of reaching the opening state as described in (Warren, 2021; Wehrens et al., 2006; Respress et al., 2012).

We implement this effect by changing parameter $k_{1}$ , (see Table 1), by ϕ . This modifies the rate of opening, and when ϕ>1 it increases the open probability. So, the updated version of the parameter $k_{1}$ reads:

$$k_{1}=\phi \cdot \frac{k_{1}^{\prime }}{k_{\mathrm{casr}}}.$$ (19)

And, finally, Phospholamban (PLB) is a regulatory protein that inhibits the activity of the Sarcoplasmatic reticulum calcium ATPase (SERCA). PKA phosphorylation of PLB decreases SERCA inhibition, which results in faster calcium recapture (Tomek and Zaccolo, 2023). The Sarcoendoplasmic reticulum calcium ATPase (SERCA) uptake reads

$$I_{up}=\frac{V_{\mathrm{maxup}}}{1+{\left(K_{up}/\phi \right)}^{2}/{{\left[Ca\right]}_{i}^{2}}_{\mathrm{free}}},$$ (20)

where the effective term, $K_{up}/\phi$ , takes into account different possible affinities resulting from PKB regulation.

Notice that, in this way, all the changes produced by phosphorylation are introduced in ϕ in Equations 18–20 as a single parameter, changing the three key targets of phosphorylation. More complex relations could be established by changing the effect in each case, but we propose that a common value is physiologically relevant and a very clear way to investigate different phosphorylation levels.

In this manuscript, we consider two phopshorylation level scenarios relative to control (ϕ=1) . In scenario 1, parameter ϕ was reduced by 50% (i.e., ϕ=1↦ϕ=0.5 ) representing a drop in phosphorylation. In scenario 2, the same parameter was increased by 100% (i.e., doubled: ϕ=1↦ϕ=2 ) representing an increase in phosphorylation.

### 2.6 Summary of computational experiments

As described in the previous sections, this study introduces a stochastic Markov Chain (MC) model in which the cell is represented by a single Scalable Aggregate Calcium Release Unit (SA-CaRU). In the limit of a large number of L-type calcium channels (LCCs) and ryanodine receptors (RyRs), the model behavior converges to that of the deterministic system. However, in finite and physiologically relevant channel numbers, the stochastic formulation captures variability in calcium fluxes, particularly in the L-type calcium current $\left(I_{\mathrm{CaL}}\right)$ and the release current from the sarcoplasmic reticulum $\left(I_{\mathrm{rel}}\right)$ .

To investigate how many channels are required for the system to approximate the deterministic limit and to quantify how calcium dynamics vary between stochastic and deterministic-like regimes, we conducted numerical simulations in which the number of LCCs in the SA-CaRU was systematically varied. Specifically, we explore $N_{\mathrm{LCC}}\in \left\{1,2,4,\cdots \;,32768\right\}$ , increasing the count by powers of two (i.e., up to 2^15^).

The purpose of this scaling was to evaluate how the degree of channel clustering affects the emergence of coordinated calcium signaling. To isolate this effect, we maintained a constant ratio between LCCs and RyRs throughout all simulations. Based on anatomical and functional estimates from the literature (Bers and Stiffel, 1993; Qu et al., 2022), we fixed $N_{\mathrm{RyR}}=5\cdot N_{\mathrm{LCC}}$ for each experiment.

For each combination of $N_{\mathrm{LCC}}$ and $N_{\mathrm{RyR}}$ , we simulated 1100 pacing cycles at a frequency of 1 Hz. The initial 100 beats were discarded to eliminate transient dynamics and ensure that the system reached steady-state conditions. The remaining 1000 beats were used for analysis. This setup enabled us to capture the beat-to-beat variability intrinsic to the stochastic formulation and to study its dependence on the channel population size.

To quantify the degree of stochasticity in our simulations, we focused on the calcium release current from the sarcoplasmic reticulum, $I_{\mathrm{rel}}$ , representing the flux of calcium ions into the cytosol. For each beat, we computed the total calcium released over a 1-s interval by summing $I_{\mathrm{rel}}\left(t\right)$ , yielding a scalar quantity that characterizes the amplitude of release. Mathematically, it reads

$$\bar{S}\left(I_{\mathrm{rel}}\right)=\int_{0}^{T}I_{\mathrm{rel}}dt=\frac{T}{N}\overset{i=N}{\underset{i=1}{\sum }}I_{\mathrm{rel}}\left(t_{i}\right)$$ (21)

where N in Equation 21 is the number of Δt steps used to simulate one period.

This summation was performed for each of the 1000 beats simulated per channel configuration, producing a distribution of $\bar{S}\left(I_{\mathrm{rel}}\right)$ values. We used descriptive statistics to characterize the variability of this distribution and to compare the degree of stochasticity between simulations with different numbers of LCCs and RyRs.

To visually assess the variability in calcium release under different channel configurations, we generated histograms of the summed release signal $\bar{S}\left(I_{\mathrm{rel}}\right)$ for each stochastic simulation. These histograms capture the distribution of calcium release amplitudes across 1000 steady-state beats for each $N_{\mathrm{LCC}}$ , and reveal how stochastic fluctuations evolve as the number of channels increases. These visualizations offer qualitative insight into the degree of stochasticity present in the system and complement the statistical descriptors of variability.

To contrast this behavior with the idealized deterministic regime, we also analyzed the last 10 pacing cycles of the deterministic version of the model. For each of these cycles, we extracted the temporal evolution of key variables, including membrane potential V , intracellular calcium concentration ${\left[Ca\right]}_{i}$ , subspace calcium ${\left[Ca\right]}_{ss}$ , L-type calcium current $I_{\mathrm{CaL}}$ , and calcium release current $I_{\mathrm{rel}}$ . These traces reflect the steady-state behavior of the system in the absence of stochastic fluctuations and serve as a visual reference against which the effects of noise can be qualitatively assessed.

The output data were produced for each of the five simulation scenarios investigated: the baseline physiological condition, the high and low phosphorylated state (representing β -adrenergic stimulation) and the LCC up and downregulation mimicking voltage-dependent modulation. This allowed for direct comparisons across pathophysiological conditions and cluster sizes.

## 3 Results

The results are organized as follows. First, we present the dynamics of the pulses for the standard configuration of the model as a function of the stochasticity, which is controlled by the size of the SA-CaRU.

Next, we introduce two modifications to the model, which enable the extraction of information not only from the deterministic dynamics, as is standard in scientific research, but also from the dependence on stochasticity under various scenarios of LCC malfunction and phosphorylation.

### 3.1 Dependence of the dynamics on the number of channels in our baseline model

#### 3.1.1 Large number of channels–deterministic limit

**FIGURE 4:**
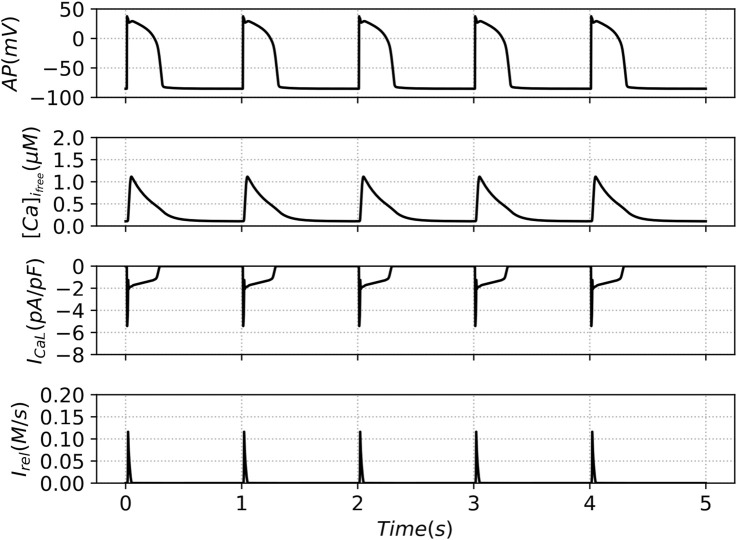
Output curves of the Action Potential (AP), Free Intracellular Calcium Transient $\left({{\left[Ca\right]}_{i}}_{\mathrm{free}}\right)$ , L-type Calcium Current $\left(I_{\mathrm{CaL}}\right)$ and Ryanodine Receptors calcium release $\left(I_{\mathrm{rel}}\right)$ . The results present the outputs of the last five beats of the deterministic model under the control condition.

As explained in the methodology, we numerically pace the deterministic single-cell model every one second through the channel $I_{st}$ . After the transitory behavior, we find a repetitive pattern of pulses as seen in Figure 4. The action potential has the usual shape of a ventricle cell (Ten Tusscher and Panfilov, 2006) and the free calcium and the two fluxes determined by $I_{\mathrm{CaL}}$ and $I_{\mathrm{rel}}$ take the usual shape from both descriptions based in HH-gates, see (Ten Tusscher and Panfilov, 2006) and the corresponding description in terms of Markov chains (Novaes et al., 2022). In every beat, the concentration of ions that leave RyR channels $\left(I_{\mathrm{rel}}\right)$ remains constant, reaching a steady state.

#### 3.1.2 Finite number of channels–stochastic effects

**FIGURE 5:**
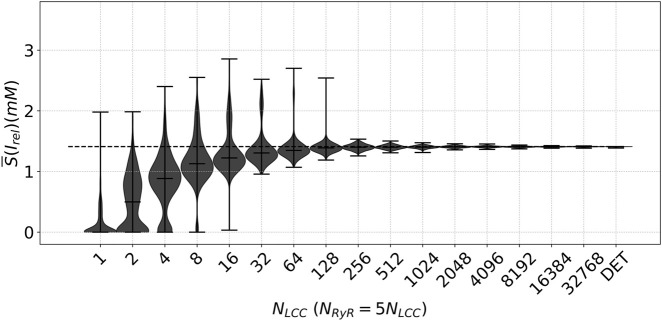
Descriptive Measures of the stochastic metric $\bar{S}$ associated with the average calcium release from the SR of the LCC channels for the control case (black line). For each N_LCC_ and, consequently, N_RyR_, there were generated n=1000 outputs. DET denotes the deterministic version of the model.

We also consider the stochastic behavior of the ion channels and employ the description in terms of a stochastic algorithm as explained in the methodology. In the limit of a large number of channels, the behavior of the system has to coincide with the deterministic simulation. For a lower number of LCC, we want to observe the type of behavior obtained. We have measured the total release of calcium through the RyR channels to visualize the different dynamics of the model as a function of the number of channels considered; see Figure 5. As expected, the release converges to the deterministic value when the number of channels is large.

**FIGURE 6:**
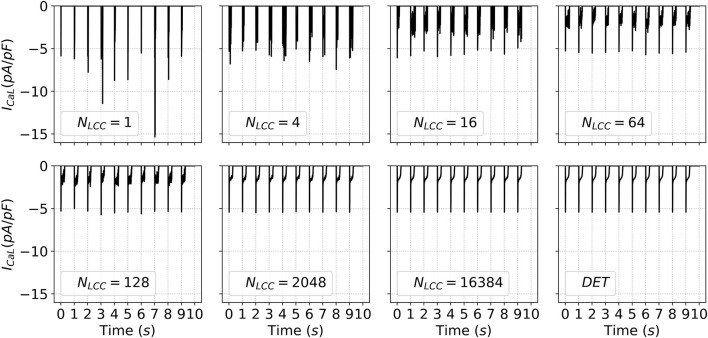
Time traces of the LCC current $I_{\mathrm{CaL}}$ during the last ten stimulation pulses. Each panel shows the temporal dynamics of $I_{\mathrm{CaL}}$ for a distinct value of $N_{\text{LCC}}$ (and, consequently, $N_{\text{RyR}}$ ), illustrating how changes in channel density modulate the calcium entrance from outside the cell. DET denotes the deterministic version of the model.

However, the release decreases as the number of channels considered is reduced. For a small number of channels, the average release is smaller and has some variability, as shown by the intervals in Figure 5. Therefore, as expected for a stochastic process, the release of calcium through RyR channels varies from beat to beat. However, the high dispersion of transients at a low number of LCC gradually converges to a Gaussian-type dispersion of values as the number of LCC increases. A remarkable result is that this dispersion is drastically reduced after $N_{\mathrm{LCC}}=128$ , where the values converge very rapidly to the deterministic case. The same reduction in the variability is observed in the $I_{\mathrm{CaL}}$ traces plotted in Figure 6. This indicates that if calcium diffusion can coordinate microdomains of up to 200 channels, or around 50 microdomains if we consider that there are 2-8 LCC per cluster, the local coupled-behavior does not exhibit strong stochasticity.

**FIGURE 7:**
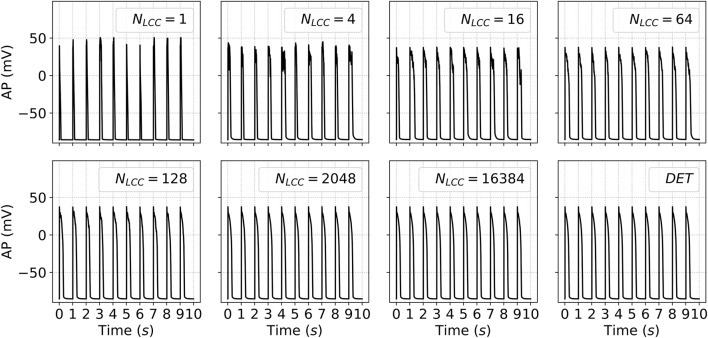
Time traces of the APs during the last ten stimulation pulses. Each panel shows the temporal dynamics of voltage for a distinct value of $N_{\text{LCC}}$ (and, consequently, $N_{\text{RyR}}$ ), illustrating how changes in channel density change the action potential. DET denotes the deterministic version of the model.

Similar tendencies to the results shown in Figure 5 have been obtained when studying the AP traces (Figure 7). One should be careful here, since the action potential in an SA-CaRU does not accurately reflect a realistic action potential. For an SA-CaRU with a large number of channels, it indeed approximates the mean-field AP, allowing us to assess whether the stochastic behavior converges to the deterministic one. For low (2–16) or intermediate (32–2024) numbers of LCC, however, the action potential is merely a representation of the local potential in the model and must not be understood as a realistic, measurable local potential. Its variability and how it differs from the deterministic gives us a clear picture of when the stochastic effect diminishes. Action Potential Duration at 50% $\left(APD_{50}\right)$ and 90% $\left(APD_{90}\right)$ are plotted as functions of the number of LCC channels, see, respectively, Supplementary Figures S1, S2. The complete set of action potential shapes and their corresponding average shapes is shown in Supplementary Figure S3 for several values of the number of LCC channels. The variability of APD and its convergence to the deterministic case are clearly observed.

**FIGURE 8:**
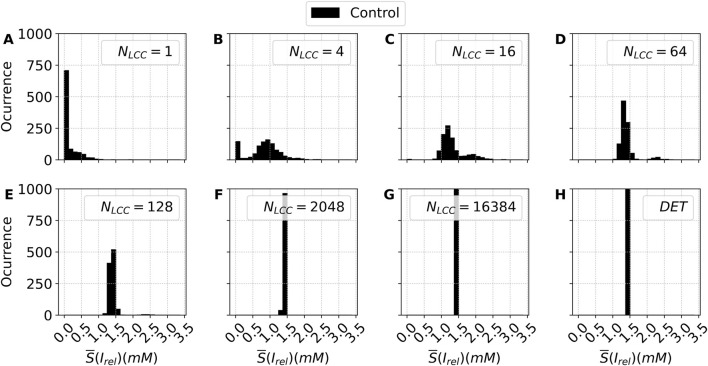
Histograms of the stochastic metric *S*– associated with the average calcium release from the SR, obtained for the control case (black bars). Each panel shows the distribution of *S*– for a distinct value of $N_{\text{LCC}}$ (and, consequently, $N_{\text{RyR}}$), illustrating how changes in channel density affect the stochastic behavior of calcium release. For each condition, n=1000 stochastic outputs were generated. Panels **(A–H)** show increasing values of $N_{\text{LCC}}$ . DET denotes the deterministic version of the model.

This is one of the most interesting features we unveil in this manuscript. The transition towards deterministic values is not a smooth transition but a sudden change in the level of noise-induced dispersion at rather low values of LCC channels. In order to better visualize this point and observe the distribution of release for the different pulses, we plot the histograms of this value for the 1000 pulses considered in each simulation for some selected examples of LCC channels in Figure 8 (baseline $N_{\mathrm{RyR}}=5N_{\mathrm{LCC}}$ ). The histograms allow for a clearer understanding of the dependence of the distribution on the number of channels. For a very low number of channels $\left(N_{\mathrm{LCC}}=1-2\right)$ , the majority of pulses do not arrive to release a substantial amount of ions, and the value is frequently zero. For a slightly high number of channels, the release increases, although it does not arrive at the deterministic limit. The distribution shows a Gaussian-like distribution around the average number, typical for a stochastic algorithm. For $N_{\mathrm{LCC}}=16$ a slightly bimodal distribution is observed, with a small second maximum above the value of the deterministic limit. The first maximum drift to the right as a function of the number of channels in a continuous displacement towards the value in the deterministic case. Crucially, around $O\left(10^{2}\right)$ channels (in the baseline scaling $N_{\mathrm{RyR}}=5N_{\mathrm{LCC}}$ ), the distribution becomes very sharp and centered on the value of the deterministic case. The implications of this finding will be discussed in the discussion.

We further assessed whether this sharp transition depends on the baseline choice of the RyR:LCC scaling. While the main results use $N_{\mathrm{RyR}}=5N_{\mathrm{LCC}}$ , we repeated the same analysis for several ratios $N_{\mathrm{RyR}}/N_{\mathrm{LCC}}\in \left\{1,2,4,8,16\right\}$ . The sharp collapse of dispersion persists across all ratios (Supplementary Figure S4), indicating that the existence of a threshold-like transition is a robust feature of the SA-CaRU dynamics rather than a consequence of a specific fixed ratio. Moreover, when the same results are represented as a function of $N_{\mathrm{RyR}}$ (Supplementary Figure S5), the curves collapse more tightly, suggesting that the effective number of RyR channels is a primary determinant of the dispersion of the SR release statistic.

**FIGURE 9:**
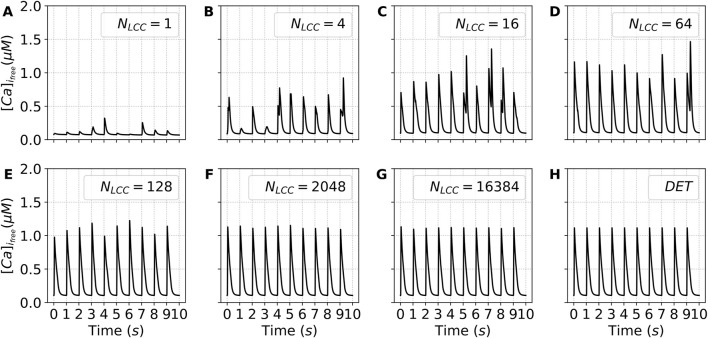
Time traces of the free cytosolic calcium concentration ( ${\left[Ca\right]}_{i_{\text{free}}}$ ) during the last ten stimulation pulses. Each panel shows the temporal dynamics of ${\left[Ca\right]}_{i_{\text{free}}}$ for a distinct value of $N_{\text{LCC}}$ (and, consequently, $N_{\text{RyR}}$ ), illustrating how changes in channel density modulate the cytosolic calcium signals. Panels **(A–H)** show increasing values of $N_{\text{LCC}}$ . DET denotes the deterministic version of the model.

In Figure 9 we show the calcium pulses along time for different values of LCC channels. For intermediate values of the number of channels, see $N_{\mathrm{LCC}}=256$ in Figure 9, the behavior resembles the deterministic case, a sequence of pulses with a relatively constant shape. For lower values of the number of channels, second pulses appear randomly along the sequence of pulses, giving rise to the bimodal distributions observed for $N_{\mathrm{LCC}}=16-64$ in Figure 8 further decrease of the number of channels produces the alternance of typical pulses with a size similar to that observed in the deterministic case, and very small pulses. Such behavior reminds us of the calcium alternans although without its typical periodicity.

### 3.2 Regulation due to modifications on LCC inactivation rates

#### 3.2.1 Large number of channels–deterministic limit

**FIGURE 10:**
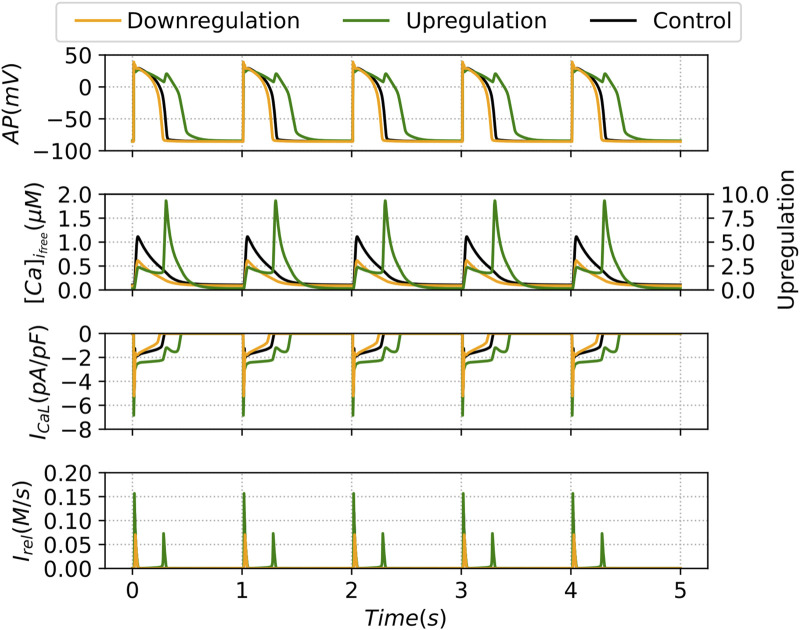
Output curves of the Action Potential (AP), Free Intracellular Calcium Transient $\left({{\left[Ca\right]}_{i}}_{\mathrm{free}}\right)$ - note the different scale for the upregulation case, L-type Calcium Current $\left(I_{\mathrm{CaL}}\right)$ and Ryanodine Receptors calcium release $\left(I_{\mathrm{rel}}\right)$ . The results present the outputs of the last five beats of the deterministic model under the decreasing (green line) and increasing (orange line) parameter η , corresponding to up and downregulation, respectively, of the LCC channels, in comparison with the control case (black line).

The up and downregulation of the LCCs will increase and decrease, respectively, the influx of calcium and therefore free calcium; as shown in Figure 10.

For upregulation of the LCC, the deterministic simulation of the whole-cell model, reducing the parameter η by 50%, shows a second peak of flux due to $I_{\mathrm{rel}}$ ; see Figure 10. The second peak, together with an increase in the release in the first peak compared to the control case, doubles the calcium release due to $I_{\mathrm{rel}}$ . This second peak of calcium release produces a secondary peak in the action potential, which lengthens the repolarization process.

The increase in the parameter η in the deterministic whole-cell model corresponds to a downregulation of the LCC channel, and produces a reduction in calcium release and in the free calcium available in the cell; as shown in Figure 10. The release resulting from flux $I_{\mathrm{rel}}$ is smaller than in the control case. However, the action potential remains similar to that in the control case.

#### 3.2.2 Finite number of channels–stochastic effects

**FIGURE 11:**
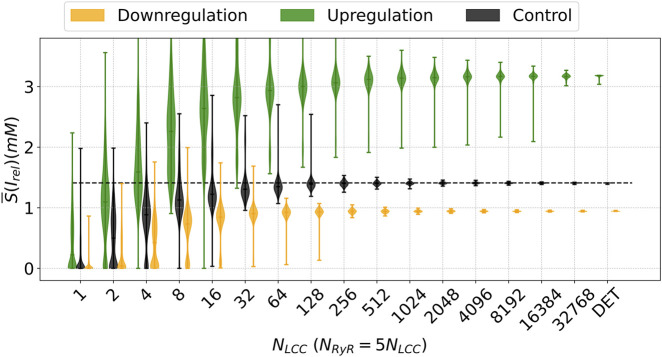
Descriptive Measures of the stochastic metric $\bar{S}$ associated with the average calcium release from the SR under the decreasing (green line) and increasing (orange line) parameter η , corresponding to up and downregulation, respectively, of the LCC channels, in comparison with the control case (black line). For each N_LCC_ and, consequently, N_RyR_, there were generated n=1000 outputs. In contrast, there are presented also the $\bar{S}$ values for the control model (CTR). DET denotes the deterministic version of the model.

When the individual behaviors of the SA-CaRU are considered, we observe a much richer phenomenon. As expected, for a very large number of channels, we recover the same dynamics of the deterministic limit. If we incorporate the stochasticity from the GRCU we obtain similar dynamics for the downregulation case; see Figure 11, but with smaller values of the total release; see Figure 11. We also obtain a slight increase in dispersion up to $N_{\mathrm{LCC}}=128$ in comparison with the control case.

For the upregulation case, we find very broad distributions up to $N_{\mathrm{LCC}}=16384$ channels; see Figure 11. This is substantially different from the previous case and indicates that, under such circumstances, the stochastic models produce different dynamics than the corresponding deterministic case.

Similar tendencies to the results shown in Figure 11 were obtained for $APD_{50}$ and $APD_{90}$ , which are plotted as functions of the number of LCC channels in Supplementary Figures S6, S7. The complete set of different shapes of the action potential and its corresponding average shape can be observed for the upregulated in Supplementary Figure S8 and downregulated cases Supplementary Figure S9, for several values of the number of LCC channels. While the downregulated case converges rapidly to the deterministic case, the upregulated case has broad distributions for a finite but large number of channels, in correspondence with the results for the calcium release observed in Figure 11.

**FIGURE 12:**
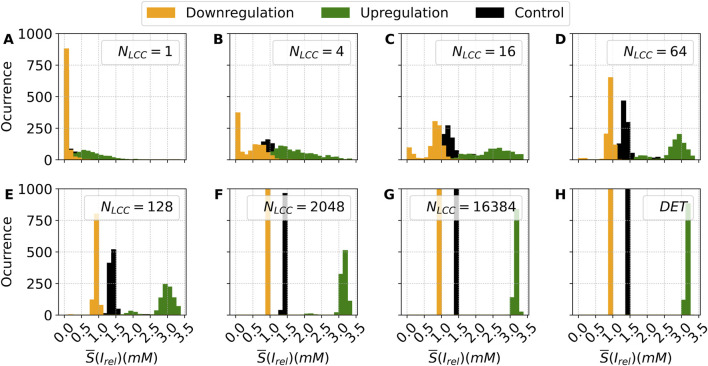
Histograms of the stochastic metric *S*– associated with the average calcium release from the SR, obtained under decreasing (green bars) and increasing (orange bars) values of the parameter η , corresponding to the up and down regulation, respectively, of the LCC channels, in comparison with the control case (black bars). Each panel shows the distribution of *S*– for a distinct value of $N_{\text{LCC}}$ (and, consequently, $N_{\text{RyR}}$ ), illustrating how changes in channel density affect the stochastic behavior of calcium release. For each condition, n=1000 stochastic outputs were generated. The distribution for the control model (CTR) is also shown for reference in each subplot. Panels **(A–H)** show increasing values of $N_{\text{LCC}}$ . DET denotes the deterministic version of the model.

The distributions of the stochastic metric $\bar{S}$ shown in Figure 11 can be very broad, depending on the number of channels. In Figure 12 we show the corresponding histograms showing the shape of the distribution of the release values for different numbers of channels, for the two cases under consideration in this section, compared to the control case.

For a small number of channels, the distributions are broad, covering the entire spectrum of values. For $N_{\mathrm{LCC}}=16$ both regulation cases show a bimodal distribution. For intermediate values, while the downregulation case gives rise to localized standard distributions around a clear value, the upregulation case keeps the bimodal distribution until large values of the number of channels, see the case $N_{\mathrm{LCC}}=2048$ in Figure 12. The broad distributions observed in Figure 11 for the upregulation case can be associated with the effects of the bimodal distributions obtained in Figure 12. Finally, for more than two thousand channels, in the three cases, the distribution collapses into a single sharp peak, recovering the deterministic cases.

**FIGURE 13:**
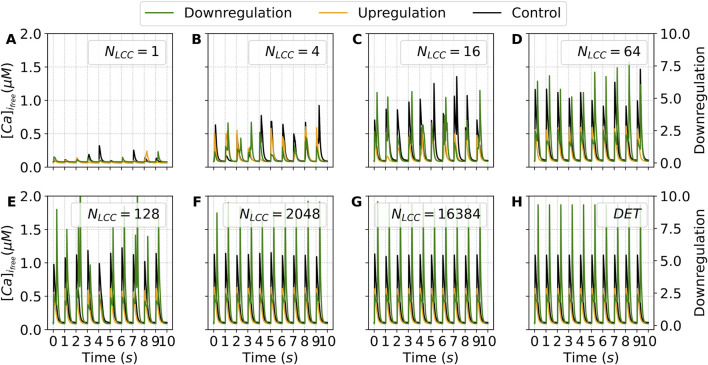
Time traces of the free cytosolic calcium concentration ( ${\left[Ca\right]}_{i_{\text{free}}}$ ) during the last ten stimulation pulses, obtained under decreasing (green lines) and increasing (orange lines) values of the parameter η , corresponding to the up and down regulation, respectively, of the LCC channels. Each panel shows the temporal dynamics of ${\left[Ca\right]}_{i_{\text{free}}}$ for a distinct value of $N_{\text{LCC}}$ (and, consequently, $N_{\text{RyR}}$ ), illustrating how changes in channel density modulate the cytosolic calcium signals. Control traces (black lines, CTR) are included for comparison. Panels **(A–H)** show increasing values of $N_{\text{LCC}}$ . DET denotes the deterministic version of the model.

Calcium release depends on the number of channels under consideration, and the resulting free calcium is different for the particular time window and the number of channels. In Figure 13, the free calcium is plotted for ten pulses for both regulatory cases and compared to the control case.

For just one single channel, see Figure 13A, the free calcium in the three cases is very small compared to the deterministic case, see Figure 13F.

For an increasing number of channels, the downregulation case shows an alternation between a small and a large peak of calcium. Finally, for $N_{\mathrm{LCC}}$ larger than 32, the simulations of the downregulation condition show a stationary shape of the calcium peak similar to the peak in the deterministic case, however, substantially smaller.

For an increasing number of channels, the upregulation case shows, as expected, much larger calcium pulses, and the shape of the pulses presents several peaks associated with multiple releases of calcium. The number of peaks is not constant for an intermediate number of channels and only collapses to a fixed value for the deterministic case, giving rise to the fixed value of $\bar{S}$ shown in Figure 11. The presence of such multiple peaks in the same calcium pulse explains the bimodal distribution found in the results shown in Figure 12 for large values of the number of channels and the corresponding broad distributions shown in Figure 11.

### 3.3 Phosphorylation

#### 3.3.1 Large number of channels–deterministic limit

We consider high and low phosphorylation by increasing and decreasing the parameter ϕ in the deterministic model, respectively.

**FIGURE 14:**
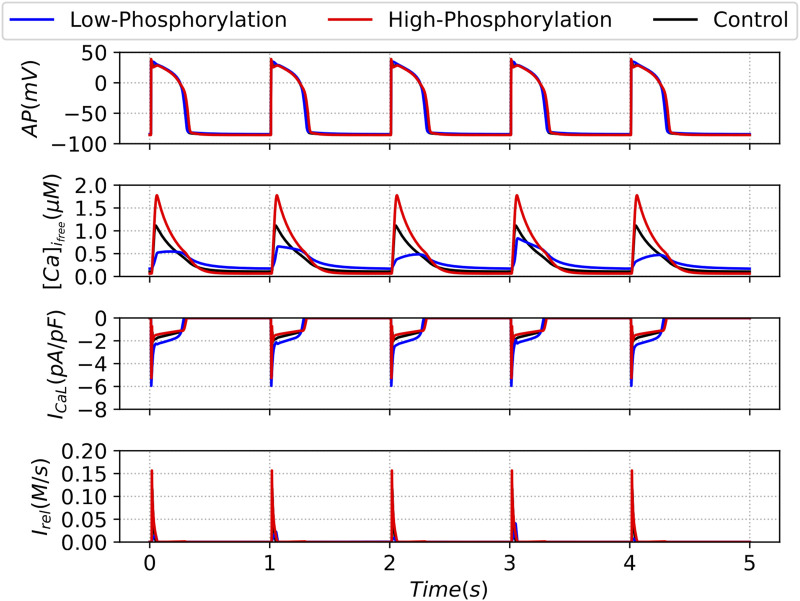
Output curves of the Action Potential (AP), Free Intracellular Calcium Transient $\left({{\left[Ca\right]}_{i}}_{\mathrm{free}}\right)$ , L-type Calcium Current $\left(I_{\mathrm{CaL}}\right)$ and Ryanodine Receptors calcium release $\left(I_{\mathrm{rel}}\right)$ . The results present the outputs of the last five beats of the deterministic model under the decreasing (blue line) and increasing (red line) parameter ϕ , corresponding to up and downregulation, respectively, of the Phosphorylation effects, in comparison with the control case (black line).

For high phosphorylation, the deterministic simulation shows an increase in the flux from the RyR channels and an increase in the free calcium after each pulse; see Figure 14. However, the action potential does not change substantially. In this case, the release is the same for all the pulses, and they are identical during the whole simulation.

The decrease in the parameter ϕ produces some unexpected effects in the deterministic dynamics of the action potential and the ion currents. Although the action potential does not seem to change in comparison with the control case, see Figure 14, the flux $I_{\mathrm{rel}}$ , and in particular the free calcium concentration, show a strong diversity of pulses with clearly different values. These calcium alternans can have important implications on contractibility. Such an alternation of values in the flux from the RyR gives rise to nonhomogeneous values of release. Therefore, in this case, the release value is not a simple value but a distribution of values. Our results on the effect of phosphorylation on cardiac intracellular calcium handling and the link to cardiac alternans corroborate with previous observations on cardiac tissue (Laurita and Rosenbaum, 2008).

#### 3.3.2 Finite number of channels–stochastic effects

**FIGURE 15:**
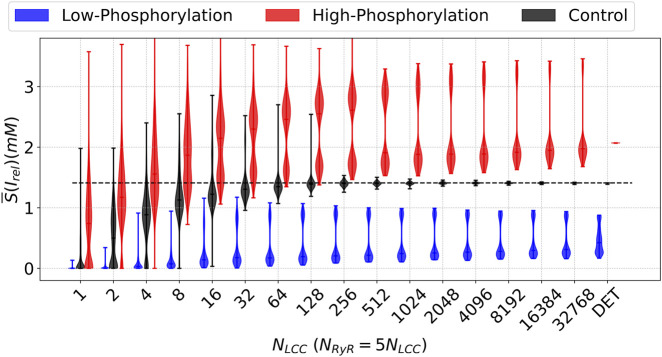
Descriptive Measures of the stochastic metric $\bar{S}$ associated with the average calcium release from the SR under the decreasing (blue line) and increasing (red line) parameter ϕ , corresponding to down and upregulation, respectively, of the Phosphorylation effects, in comparison with the control case (black line). For each N_LCC_ and, consequently, N_RyR_, there were generated n=1000 outputs. In contrast, there are presented also the $\bar{S}$ values for the control model (CTR). DET denotes the deterministic version of the model.

When stochastic simulations are included through the SA-CaRU, the dynamics change strongly with respect to the control case; see Figure 15. In both cases, low and high phosphorylation, the distributions are broad for a wide range of numbers of channels. In the case corresponding to high phosphorylation, the distributions are very broad until $N_{\mathrm{LCC}}=32768$ , and only collapse to a single peak for the deterministic case. It is important to stress that such a large coupling is unrealistic. In order to have thousands of LCC channels coupled, it would require that calcium diffusion strongly couple microdomains at very large distances within the cell. It has been well established that diffusion alone cannot synchronize this behavior. As we discuss later, this indicates that stochasticity is important and that the reduction to the deterministic case is questionable.

On the other hand, for low phosphorylation, the simulations show, for the whole spectrum of numbers of channels, a very dispersed distribution of release values; see Figure 15. Contrary to the previous case, we obtain in the case with $N_{\mathrm{LCC}}=32768$ the same behavior as in the deterministic case. Here, whole-cell models would seem more appropriate to describe changes at the microdomain level than in the previous case.

For phosphorylation, the tendencies of the results shown in Figure 15 are relatively different from the tendencies obtained for $APD_{50}$ and $APD_{90}$ , which are plotted as functions of the number of LCC channels, see, respectively, and Supplementary Figures S10, S11. In both cases, the duration of the action potential remains closer to the control case. The complete set of different shapes of the action potential and the corresponding average shapes can be observed for the low phosphorylation case in Supplementary Figure S12 and for the high phosphorylation case in Supplementary Figure S13, for several values of LCC channels. A second peak in the action potential, eventually associated with an ectopic beat, remains for a large number of simulations, see Supplementary Figure S13.

**FIGURE 16:**
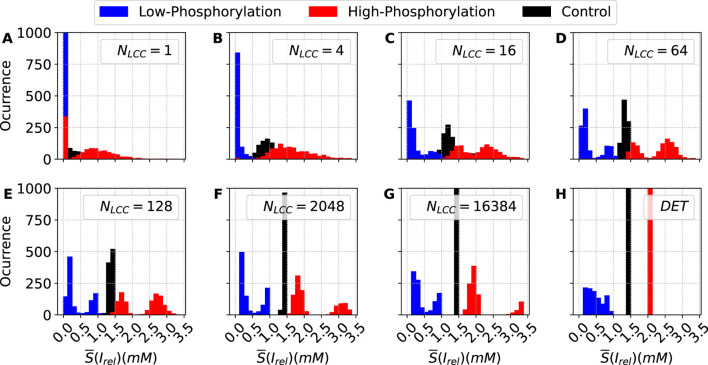
Histograms of the stochastic metric *S*– associated with the average calcium release from the SR, obtained under decreasing (blue bars) and increasing (red bars) values of the parameter ϕ , corresponding to the down and up regulation, respectively, of the phosphorylation effects, in comparison with the control case (black bars). Each panel shows the distribution of *S*– for a distinct value of $N_{\text{LCC}}$ (and, consequently, $N_{\text{RyR}}$ ), illustrating how changes in phosphorylation state affect the stochastic behavior of the average calcium release. For each condition, n=1000 stochastic outputs were generated. The distribution for the control model (CTR) is also shown for reference in each subplot. Panels **(A–H)** show increasing values of $N_{\text{LCC}}$ . DET denotes the deterministic version of the model.

The assessment of the whole-cell deterministic model can be better understood by the visualization of the histograms associated with the simulations. For stochastic simulations, the release follows a bimodal distribution with two separated maxima compared to the conditions studied above; see Figure 16. See the case with $N_{\mathrm{LCC}}=2048$ , shown in Figure 16, where both phosphorylation cases show clear bimodal distributions in comparison with the single peak of the control case.

For the case of low phosphorylation, in the deterministic limit, a continuous distribution of values is observed; see Figure 16. If the number of channels is decreased, this continuous distribution gives rise to a clear bimodal distribution between two values with a maximal probability, as a clear sign of calcium alternans.

**FIGURE 17:**
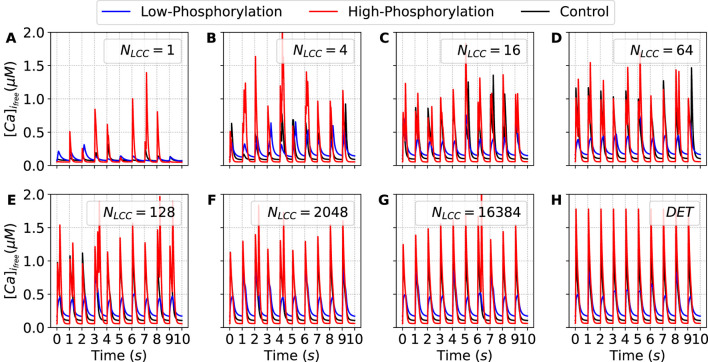
Time traces of the free cytosolic calcium concentration ( ${\left[Ca\right]}_{i_{\text{free}}}$ ) during the last ten stimulation pulses, obtained under decreasing (blue lines) and increasing (red lines) values of the parameter ϕ , corresponding to the down and up regulation, respectively, of the phosphorylation effects. Each panel shows the temporal dynamics of ${\left[Ca\right]}_{i_{\text{free}}}$ for a distinct value of $N_{\text{LCC}}$ (and, consequently, $N_{\text{RyR}}$ ), illustrating how changes in the phosphorylation state modulate the cytosolic calcium signals. Control traces (black lines, CTR) are included for comparison. Panels **(A–H)** show increasing values of $N_{\text{LCC}}$ . DET denotes the deterministic version of the model.

Figure 17 shows the calcium pulses for different numbers of LCC channels. While the bimodal distribution observed under high-phosphorylation conditions may be partly attributed to stochastic fluctuations arising from the numerical simulation, the distribution obtained under low-phosphorylation conditions reflects differences in the amplitudes of calcium pulses induced by external pacing. This behavior confirms the presence of calcium alternans across a broad range of channel numbers.

## 4 Discussions and conclusion

We have integrated an MC-based L-type current model together with a four-state stochastic RyR model into a single SA-CaRU framework for human ventricular myocytes (Novaes et al., 2022), previously fitted to a well-established model (Ten Tusscher and Panfilov, 2006). Our model maintains deterministic whole cell output at high channel counts while effectively capturing stochastic microdomain subcellular release behavior at lower channel densities. In particular, the transition from random to deterministic-like behavior occurs sharply when the number of L-type calcium channels (LCCs) in the unit exceeds the threshold of $O\left(10^{2}\right)$ LCCs.

This has significant implications for our understanding of subcellular calcium dynamics, as it clarifies the importance of effective diffusion coupling between microdomains. The coupling between the LCC and RyR microdomains is influenced by several factors, including the characteristic diffusion length, the distance between microdomains, and the amount of buffering present. Higher diffusion rates, closer microdomain proximity, and minimal buffering pathways lead to greater effective coupling. Since a single microdomain contains only a few LCCs, effective diffusion coupling that can synchronize fewer than 50 microdomains results in a high degree of subcellular variance. In this case, whole cell behavior reflects the average of thousands of highly stochastic and independent units. Conversely, if diffusion coupling can synchronize around 100 microdomains, we begin to see distinct domains of deterministic subcellular behavior. Thus, whole-cell calcium cycling can be understood in terms of the coupling of deterministic systems, both in relation to spark and wave activity.

The degree of coupling between microdomains is not arbitrary; it is physically constrained by the maximum diffusion coefficient of calcium ions in the cytosol ( ${\text{D}}_{Ca}∼800\;\mu m^{2}/s$ ). As the system size increases (i.e., a larger number of CRUs), the time required for diffusion limits the ability of the system to synchronize. Consequently, assumptions of infinite or very high coupling become physically unrealistic for large clusters, as there is a strong finite-size dependence on the effective coupling (Alvarez-Lacalle et al., 2015).

This opens an important avenue for future research. Specifically, the levels of coupling among microdomains within a heterogeneous spatial grid become very important, since the average cell outcome can depend crucially on them. The simulation of spark-induced spark activation and wave propagation in heterogeneous grids should be the next step in our analysis. The key insight from this contribution is that if deterministic behavior emerges spontaneously at around 100 channels, then whether the observed level of coupling crosses this threshold is key to understanding the framework of subcellular calcium cycling and how to transition to proper whole-cell behavior.

A spatially distributed array of CaRUs coupled by cytosolic diffusion represents the most realistic setting for spark-induced spark activation and ${\text{Ca}}^{2+}$ wave propagation. The present SA-CaRU framework provides a mean-field baseline that quantifies when stochastic dynamics converge to deterministic behavior as the effective degree of aggregation increases. Extending this work to heterogeneous CRU grids with explicit diffusion coupling is a natural next step, and will allow testing whether physiologically realistic coupling strengths can reach the effective synchronisation regime identified here.

With this in mind, we return to the interpretation of $N_{\mathrm{LCC}}$ in the SA-CaRU as an effective proxy for microdomain aggregation. An increase in the number of channels can be understood as an increase in the level of coupling among microdomains. A SA-CaRU with a few LCC reproduces the behavior of a single microdomain. Simulating a SA-CaRU of tens of thousands of LCC is basically a cell that is globally coupled by an effective infinite diffusion of intracellular ${\text{Ca}}^{2+}$ . Intermediate values represent realistic diffusion occupying tens of microdomains. By analyzing how the behavior of a single Scalable Aggregate Calcium Release Unit (SA-CaRU) evolves as the number of constituent channels increases, we can directly assess the relationship between stochastic microdomain activity and deterministic whole-cell behavior and track if there is a robust relation between deterministic (whole-cell) and the stochastic behavior obtained at a realistic level of microdomains coupling.

This analysis provides a reasonably quantitative basis for evaluating the validity of deterministic whole-cell models. Our units correctly reproduce the behavior of macrodomains when $N_{\mathrm{LCC}}$ is low. As the number of channels increases, our mean-field Calcium release Units could accurately capture the behavior of diffusion-coupled units. At very large LCC channels, the results are just a mean-field analysis that allows us to test when the whole-cell model is recovered. If these large $N_{\mathrm{LCC}}$ units do not reproduce the deterministic-like behavior, it is highly unlikely that the whole-cell deterministic model can properly reproduce the average behavior of a cluster realistically coupled with diffusion. In a sense, such models rely either on an unphysically high degree of coupling between microdomains, inconsistent with diffusion-limited calcium signaling observed in real cells, or a strongly synergetic behavior between calcium diffusion and synchronization into a single action potential. This would render the model’s outcome fundamentally different from what one would expect. In this situation, whole-cell models would not adequately describe the subcellular level at the whole-heart or tissue level.

Note that the models for RyR and LCC have been generated to reproduce whole-cell properties (Novaes et al., 2022) rather than specific single-channel recordings. Consequently, while the model captures ensemble behavior accurately, the specific dwell-time statistics and microscopic event correlations should be interpreted as a plausible mechanism consistent with global constraints, rather than a uniquely determined solution.

While the quantitative results presented here are influenced by the specific parameterization of the baseline model, the qualitative findings are primarily governed by the Calcium handling subsystem rather than background transmembrane currents. Consequently, we expect these conclusions to generalize to other human ventricular models that share similar balances of LCC, RyR, and SERCA fluxes. However, significant deviations are expected in models representing small mammals, where the much faster SERCA dynamics fundamentally alter the stability and recovery of the calcium transient.

This SA-CaRU-based approach has enabled us to investigate, under varying degrees of stochasticity, the effects of LCC upregulation and high phosphorylation. It allowed us to move beyond the conventional deterministic whole-cell analysis based solely on parameter variation, providing insight into the consequences of increased stochastic behavior. Our results show, for instance, that deterministic simulations under high phosphorylation yield qualitatively different outcomes than their stochastic counterparts, even when a very large number of channels is considered, indicating noise-sustained switching between distinct response regimes. Likewise, stochastic simulations under LCC upregulation display substantial variability even for large channel populations, calling into question the reliability of deterministic whole-cell model predictions in such regimes.

A key mechanistic insight behind this non-convergence is that, under these pathological protocols, the coupled voltage–calcium subsystem operates close to a high-gain threshold, so that stochastic fluctuations can induce *beat-to-beat switching* between distinct response regimes. In practical terms, individual beats can fall either into a low-release trajectory (weak CICR recruitment) or into a high-release trajectory (strong CICR recruitment), producing non-Gaussian and often bimodal release statistics. This switching is also reflected in the action potential morphology. Under LCC upregulation, the deterministic model already exhibits an EAD/secondary dome, and the stochastic AP ensemble converges only at very large $N_{\mathrm{LCC}}$ toward this same EAD-like morphology (Supplementary Figure S8), indicating delayed convergence due to enhanced sensitivity of the repolarization phase to trigger variability. Under high phosphorylation, by contrast, the deterministic AP does not display an EAD-like feature, yet a small late repolarization shoulder persists in a subset of stochastic realizations across a wide range of $N_{\mathrm{LCC}}$ (Supplementary Figure S13). This shows that stochasticity can sustain access to alternative repolarization trajectories even when the deterministic solution remains smooth, consistent with a noise-driven switching mechanism near the CICR/repolarization threshold.

There is substantial evidence that LCC dysregulation contributes to the etiology of heart failure (Khalid et al., 2025). Arrhythmic electrical remodeling, often characterized by altered channel density, increases susceptibility to sudden death in patients with heart failure (Erickson et al., 2011). Furthermore, experimental models have shown that such alterations can induce hypertrophy and heart failure in mice (Goonasekera et al., 2012; Van Oort et al., 2010). These pathological conditions, where the number of functional channels may be critical, highlight the importance of stochastic models that can accurately capture the transition between subcellular noise and whole-cell dynamics.

The results presented here may also guide the choice of discretization in mesoscopic subcellular models, depending on the problem under study. Such models do not exactly reproduce all subcellular dynamics but retain aspects of subcellular structure, typically using discretizations of about 2.5 μm (Prudat and Kucera, 2014), 8 μm (Gouvêa de Barros et al., 2015), or 15 μm (Jacquemet and Henriquez, 2009). This approach is analogous to that used in extracellular–membrane–intracellular (EMI) models (Jæger et al., 2021), which employ discretizations around 10 μm, and in Kirchhoff network models, which extend the EMI framework (Jæger and Tveito, 2023).

We should stress that our results indicate a fundamentally different behavior at lower levels of coupling, from intermediate levels up to the large-cell scale. In this regime, whole-cell models would provide a completely inaccurate picture under any realistic assumption of available coupling. This discrepancy is even more severe than other well-known mismatches between subcellular and whole-cell models discussed in the literature. For example, in the case of calcium alternans, as pacing frequencies increase, whole-cell models can reproduce the appearance of an order–disorder phase transition (Alvarez-Lacalle et al., 2015) as a period-doubling bifurcation. In that case, at least, the general behavior remains similar, although important effects may appear at the tissue level. In contrast, in the cases presented here, subcellular and diffusion-limited calcium models would produce fundamentally different outcomes from whole-cell deterministic models. One could argue that whole-cell models should not be used to reproduce whole-heart or tissue behavior under any malfunction unless an assessment of the type presented here has been previously carried out.

In conclusion, integrating the stochastic L-type calcium channel and four-state RyR models within the SA-CaRU framework provides a versatile platform for bridging subcellular stochasticity and deterministic whole-cell calcium dynamics. By explicitly quantifying how the transition from stochastic to deterministic behavior depends on the number of channels, this approach enables a systematic evaluation of the conditions under which deterministic modeling remains valid or stochastic effects become essential. The SA-CaRU-based methodology thus offers a powerful means to elucidate how microscopic fluctuations influence macroscopic calcium cycling, advancing the development of more physiologically realistic models of cardiac excitation–contraction coupling.

## Data Availability

The original contributions presented in the study are included in the article/Supplementary Material, further inquiries can be directed to the corresponding author.
