# Supplementary material for: Multiscale stochastic modeling for calcium dynamics in cardiac electrophysiology: assessing whole-cell model reliability under phosphorylation and LCC downregulation
Source: Front Netw Physiol. 2026 Apr 13;6:1727426. doi: 10.3389/fnetp.2026.1727426 (PMC13111126; doi:10.3389/fnetp.2026.1727426)
Supplement: Supplementary file 1 [file DataSheet1.pdf]

Supplementary Material

1 ACTION POTENTIAL ANALYSIS

We use the Action Potential (AP) as a whole-cell readout to link microdomain stochasticity to macroscopic electrophysiology. Because the single-GCRU model shows a sharp stochastic-deterministic shift around 100 LCCs, we assess AP duration and beat-to-beat variability across control, LCC regulation (η), and phosphorylation (ϕ) scenarios, under the same pacing/analysis protocol used for the main text results.

1.1 Methods – AP Extraction and Metrics

Single-cell simulations were paced at 1 Hz using the stimulus current I_{st}. After an initial transient of 100 beats, the subsequent 1000 beats were analyzed, following the protocol adopted in the main text. For each beat we computed Resting Membrane Potential (RMP); maximum AP value (max Vm); and APD_{50} and APD_{90}. Violin summaries are used for stochastic conditions. Note (RMP). RMP varied by $<1\%$ across all scenarios and channel counts; therefore, RMP is not shown in the Results (available upon request).

1.2 Control Condition - Deterministic Reference

APD vs. N_{LCC}. In control (black), APD_{50} (Figure S1) and APD_{90} (Figure S2) converge monotonically to the deterministic value as N_{LCC} increases. Dispersion is largest at very low N_{LCC} (1–16) and collapses as soon as the amount of N_{LCC} increases, consistent with the stochastic-to-deterministic transition.
AP waveforms. Time traces (Figure S3) show that for low N_{LCC} the AP morphology is slightly more variable (plateau thickness/timing), whereas for $N_{LCC} \geq 256$ the waveforms overlap the deterministic trace (DET panel). Peak V_m remains near control across N_{LCC}.

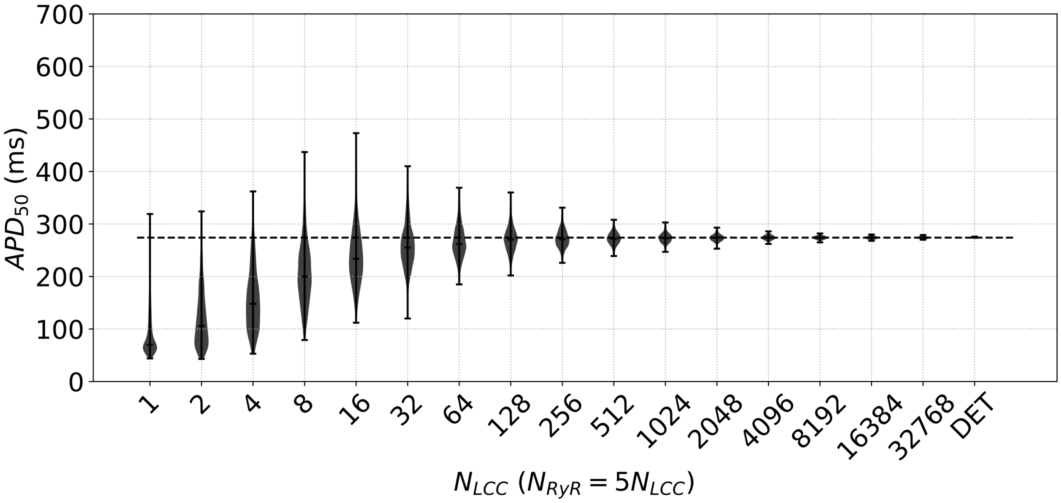

Figure S1. Values of APD_{50} are shown as violin plots across stochastic beats. Dispersion is largest for $N_{\mathrm{LCC}} \leq 16$ and collapses as soon as the amount of N_{LCC} increases, approaching the deterministic value (DET).

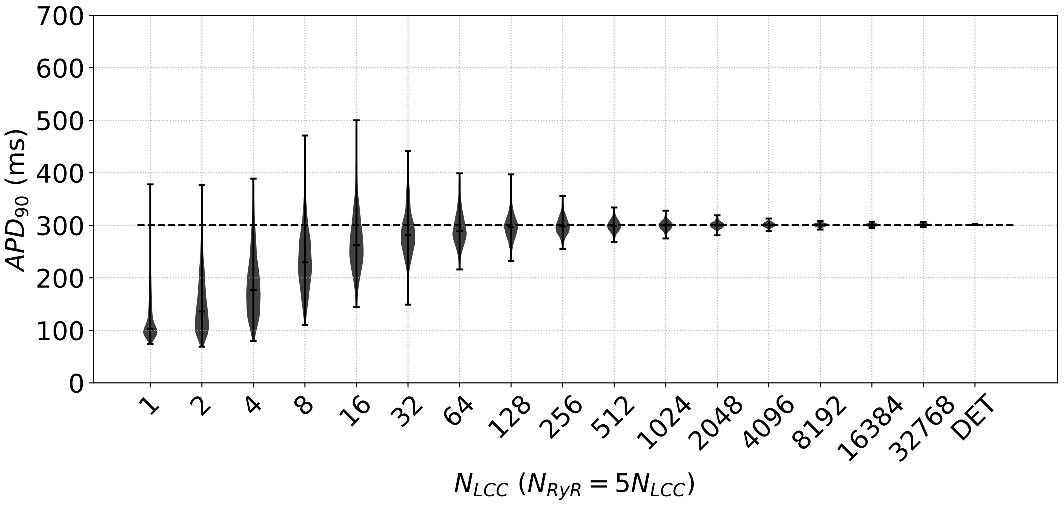

Figure S2. Values of APD_{90} are shown as violin plots across stochastic beats. Dispersion is largest for $N_{\mathrm{LCC}} \leq 16$ and collapses as soon as the amount of N_{LCC} increases, approaching the deterministic value (DET).

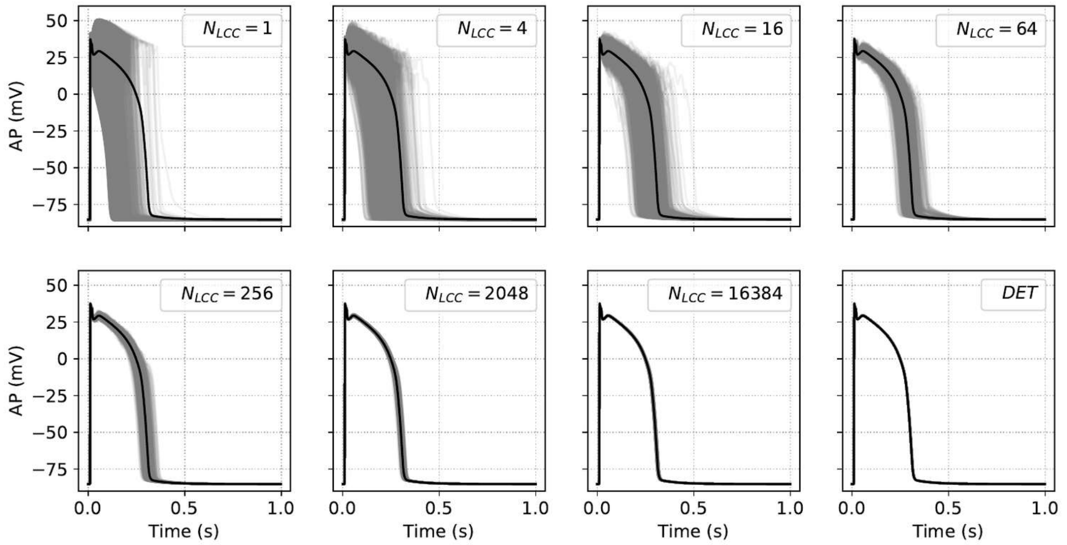

Figure S3. AP traces for representative N_{LCC} values show morphology variability at low N_{LCC} and near-perfect overlap with DET for $N_{LCC} \geq 256$. Peak V_m remains stable.

1.3 Sensitivity to the RyR:LCC scaling

In the main manuscript we fixed the relative scaling between ryanodine receptors and L-type calcium channels as

$$N_{RyR} = 5\,N_{LCC}, \tag{S1}$$

in order to reduce the parameter space and focus on the stochastic-to-deterministic transition driven by the effective channel population. Since the sharp collapse of release variability around $\mathcal{O}(10^2)$ channels is one

of the key observations of this work, here we assess its robustness with respect to the assumed RyR:LCC ratio.

To this end, we repeated the control simulations while varying the RyR:LCC ratio over the range

$$\frac{N_{RyR}}{N_{LCC}} \in \{1, 2, 4, 8, 16\}, \tag{S2}$$

keeping all other parameters unchanged. For each pair (N_{LCC}, N_{RyR}), we generated $n = 1000$ stochastic realizations and computed the same stochastic release descriptor used in the main text, namely the metric $\overline{S}$ associated with the average SR calcium release (derived from I_{rel}).

Figure S4 shows the descriptive measures of $\overline{S}$ as a function of N_{LCC} for the different RyR:LCC ratios. The qualitative behavior is robust: for low channel numbers the dispersion is large, and beyond a threshold the variability collapses rapidly toward the deterministic value. While the precise transition point shifts mildly in terms of N_{LCC} as the ratio changes, the existence of a sharp threshold-like transition is preserved across all ratios.

To better interpret which population size governs the dispersion of SR release, Fig. S5 replots the same results as a function of N_{RyR}. In this representation, the curves for different ratios collapse more tightly, suggesting that the effective number of RyR channels is a primary determinant of the dispersion of the SR release statistic, consistent with the fact that $\overline{S}$ is defined from I_{rel}.

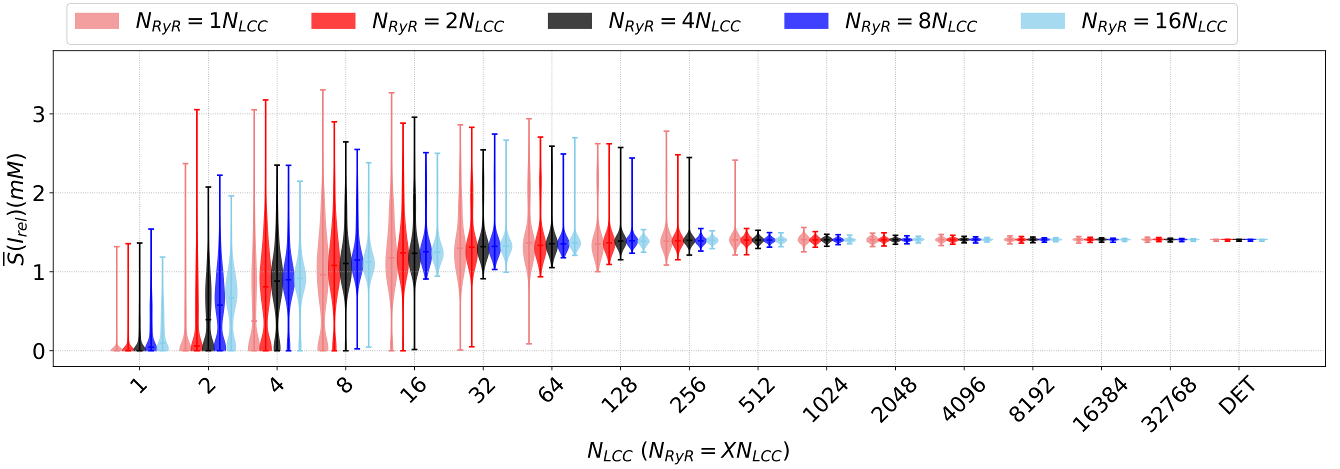

Figure S4. Sensitivity of the stochastic SR-release descriptor to the RyR:LCC scaling, shown as a function of the number of L-type calcium channels. Descriptive measures (violin plots) of the stochastic metric $\overline{S}$ associated with the average SR calcium release (derived from I_{rel}) for the control case, computed from $n = 1000$ stochastic realizations at each channel number. Different colors correspond to different RyR:LCC ratios $N_{RyR}/N_{LCC} \in \{1, 2, 4, 8, 16\}$. The deterministic reference (DET) is shown for comparison.

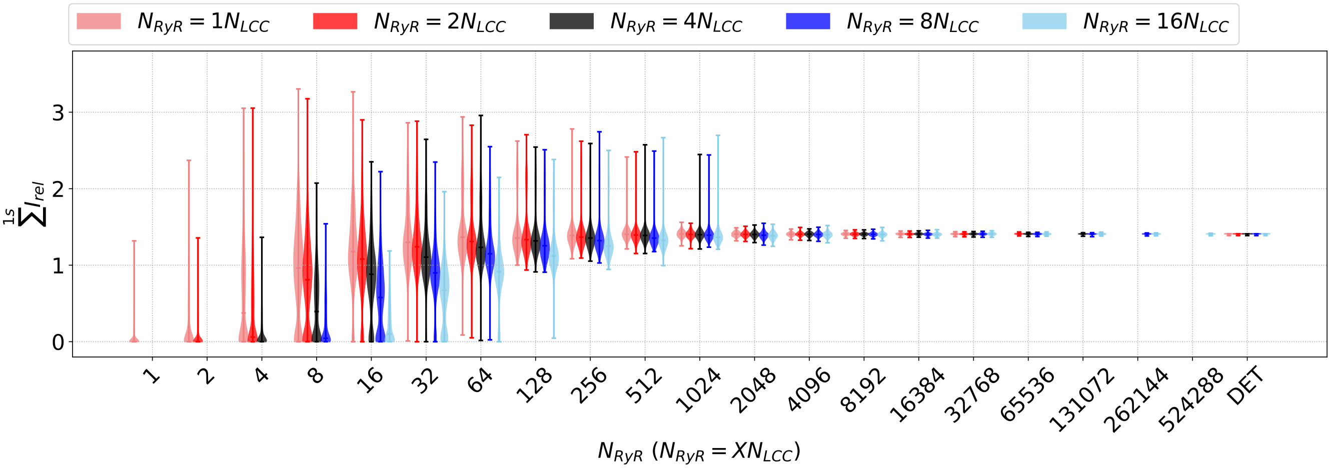

Figure S5. Same data as Fig. S4, replotted as a function of the number of RyR channels N_{RyR}. Presenting the dispersion of $\overline{S}$ against N_{RyR} yields a tighter collapse across different ratios, indicating that the effective size of the RyR population is a dominant determinant of the SR-release variability in the control condition. The deterministic reference (DET) is shown for comparison.

1.4 LCC regulation (η) – up/down-regulation

Upregulation ($\eta = 0.5$, green). Relative to control, APD_{50} and APD_{90} are up-shifted across N_{LCC} (Figures S6 and S7). The separation persists to high N_{LCC}, indicating genuine AP prolongation rather than only stochastic scatter. Waveforms (Figure S8) exhibit a subtle secondary dome/shoulder during repolarization, consistent with a second release component; variability at low N_{LCC} delays convergence.

Downregulation ($\eta = 1.5$, orange). APD curves track close to control across N_{LCC} (Figures S6 and S7), with only mild shortening tendencies. Time-traces (Figure S9) overlay control for $N_{LCC} \geq 256$, reflecting a rapid collapse of dispersion. Peak V_m remains near control in both η scenarios.

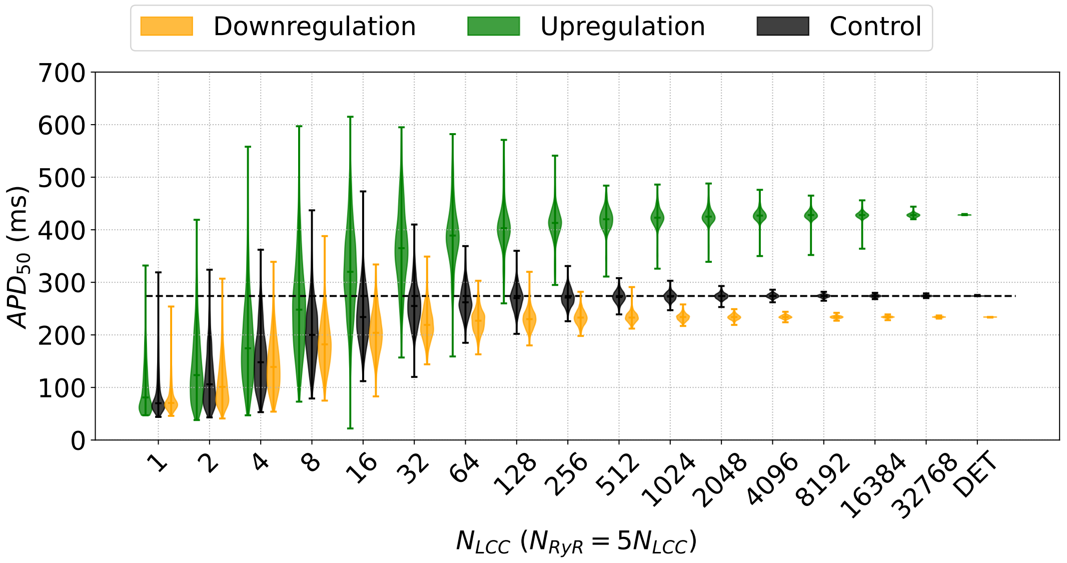

Figure S6. Upregulation ($\eta = 0.5$) shifts APD_{50} upward across N_{LCC} with broader scatter at small N_{LCC}; Downregulation ($\eta = 1.5$) tracks near control.

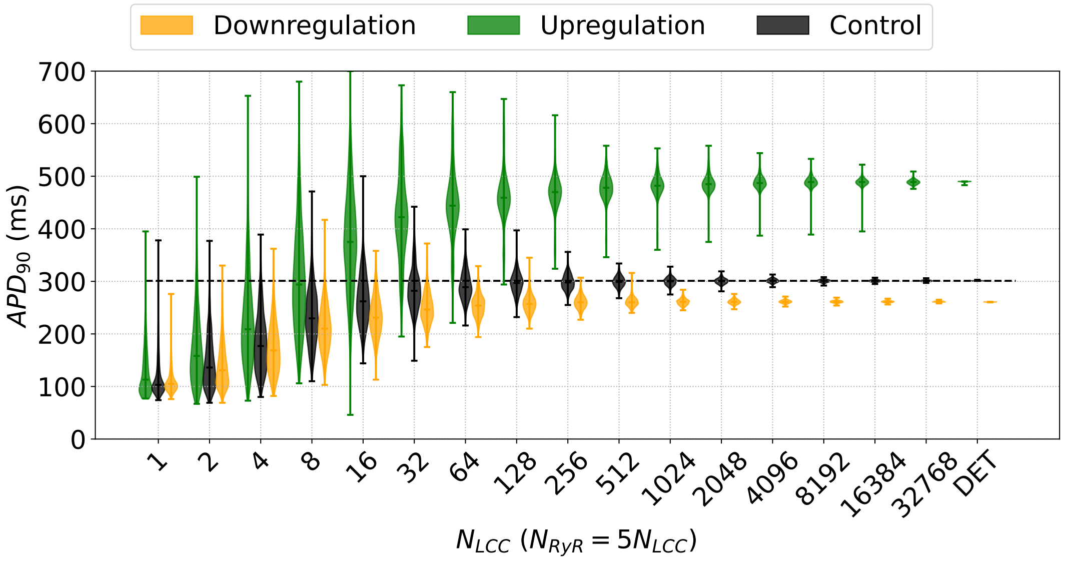

Figure S7. For APD_{90}: prolongation with $\eta = 0.5$ (Upregulation), near-control values with $\eta = 1.5$ (Downregulation).

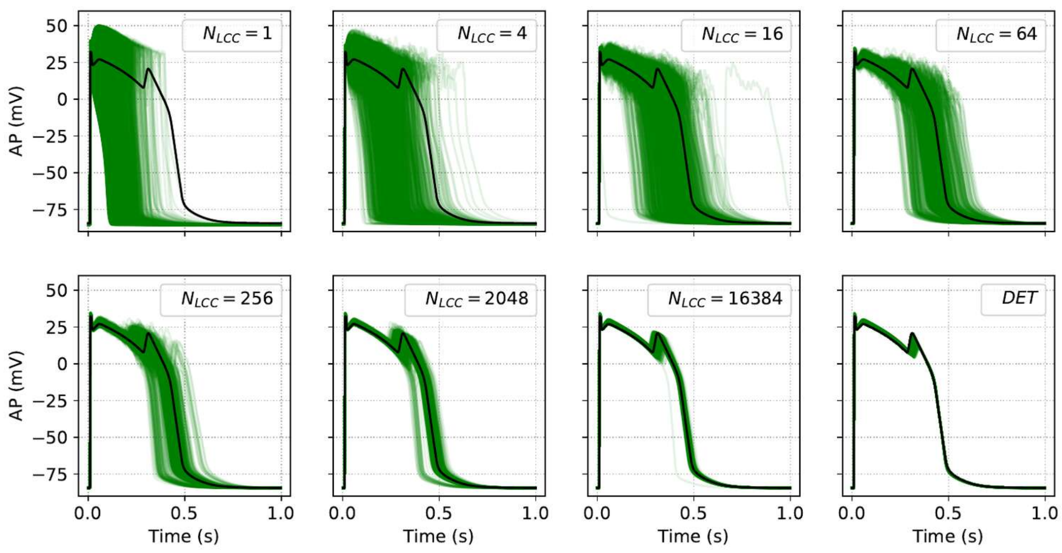

Figure S8. Upregulation ($\eta = 0.5$). Subtle secondary dome/shoulder in repolarization and prolonged AP; larger variability at low N_{LCC}, convergence at high N_{LCC}.

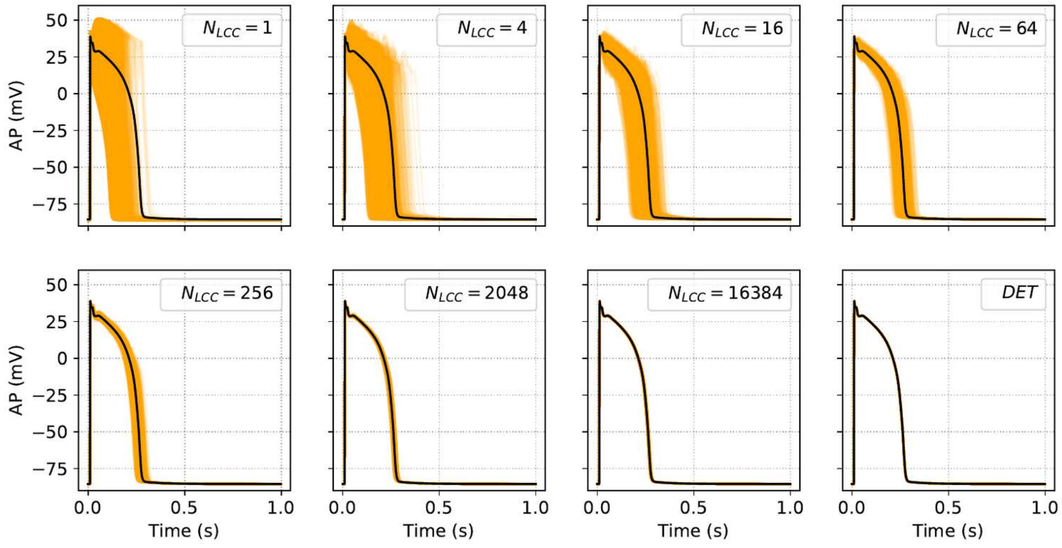

Figure S9. Downregulation ($\eta = 1.5$) Near-control morphology; rapid convergence with $N_{LCC} \geq 256$.

1.5 Phosphorylation (ϕ) – low/high

Low phosphorylation ($\phi = 0.5$, blue). APD_{50}/APD_{90} (Figures S10 and S11) remain close to control at all N_{LCC} with modest scatter at small N_{LCC}. Waveforms (Figure S12) show near-control AP shape even as calcium release increases.

High phosphorylation ($\phi = 2.0$, red). APD curves (Figures S10 and S11) exhibit larger spread and, at intermediate N_{LCC}, non-Gaussian/possibly bimodal patterns consistent with calcium alternans–like

behavior. Time-traces (Figure S13) show alternating plateau duration/thickness at low–intermediate N_{LCC}. As N_{LCC} grows, dispersion narrows but converges more slowly than in control/high-ϕ. Peak V_m remains near control.

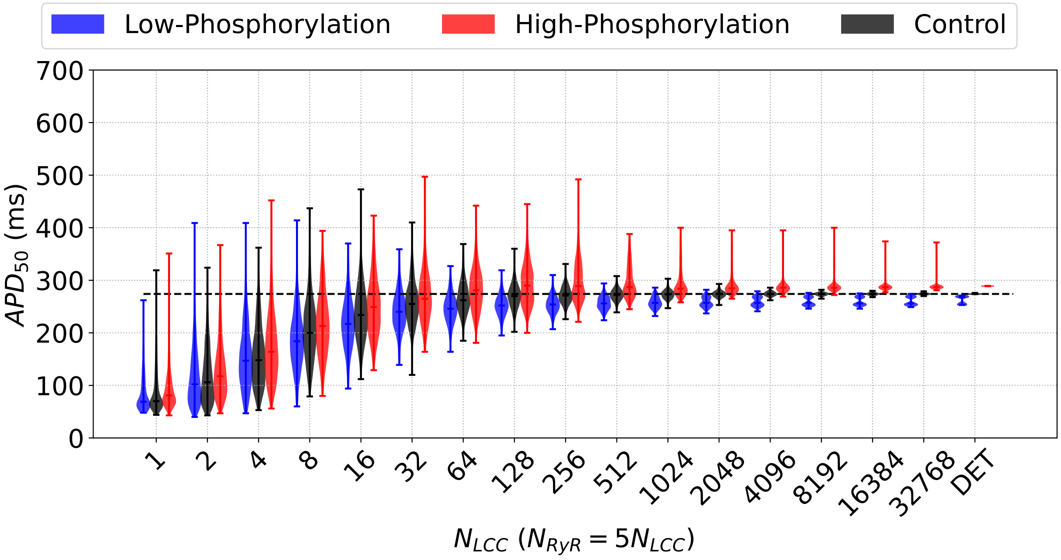

Figure S10. Low ϕ remains close to control; high ϕ shows broader dispersion and slower convergence vs. N_{LCC}.

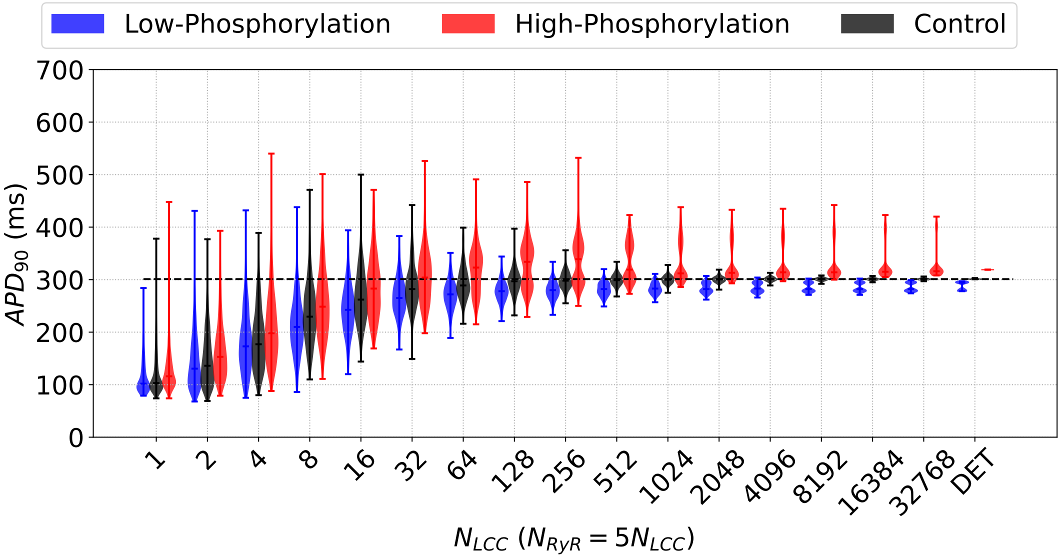

Figure S11. Same trends as APD_{50}; high ϕ can display non-Gaussian/bimodal distributions consistent with alternans-like behavior at intermediate N_{LCC}.

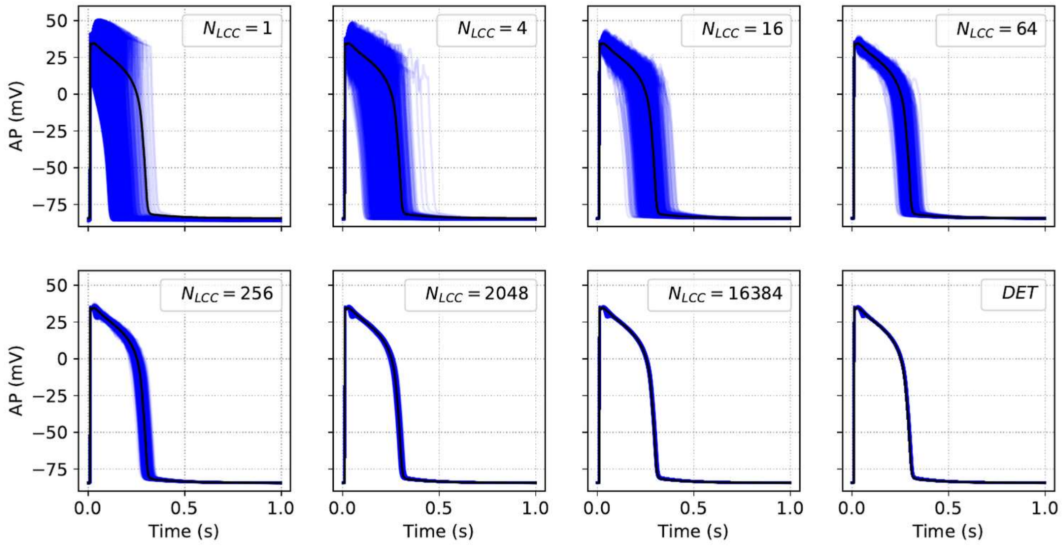

Figure S12. Near-control morphology across N_{LCC}; deterministic-like overlap at high N_{LCC}.

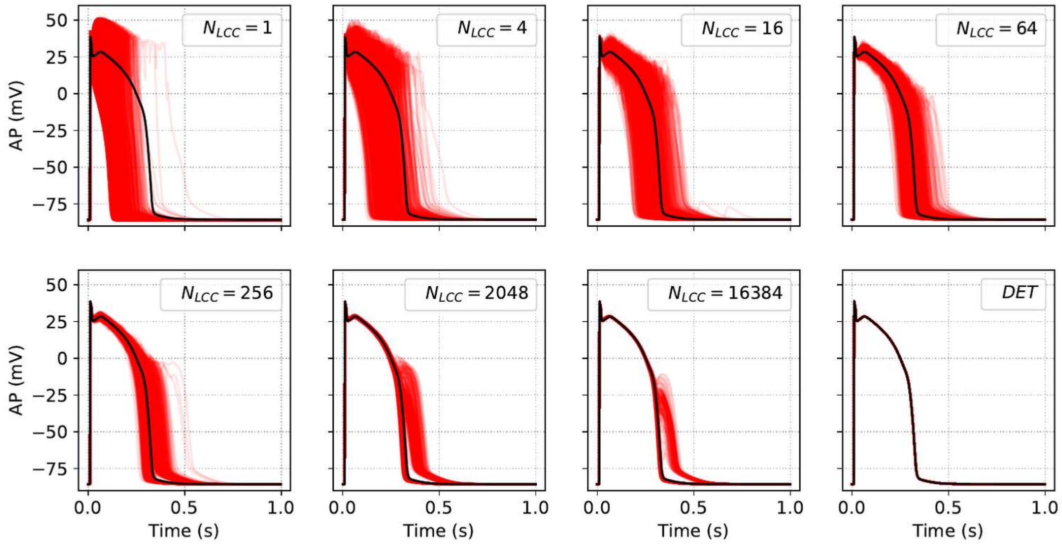

Figure S13. Alternation in plateau duration/thickness at low-intermediate N_{LCC}; variability diminishes as N_{LCC} increases but remains slower to converge than control.

1.6 Summary and Implications

Across conditions, APD dispersion shrinks with increasing N_{LCC} and stabilizes near the deterministic limit; the sharp improvement around ∼256 LCCs reflects microdomain coordination. Down-regulating LCC inactivation ($\eta = 0.5$) yields sustained AP prolongation plus waveform changes (secondary dome) that persist even at high N_{LCC}. High phosphorylation ($\phi = 2.0$) introduces alternans-like APD variability at low-intermediate N_{LCC} and slows convergence. High phosphorylation ($\phi = 0.5$) keeps APD near control. Peak V_m remains broadly stable, and RMP varies by $< 1\%$.

These AP trends corroborate the calcium-level findings: certain parameter regimes produce whole-cell AP behaviors that cannot be captured by a purely deterministic reduction unless microdomain coordination is unrealistically strong.

2 DETERMINISTIC EQUATIONS FOR I_{CaL} AND I_{rel} MC

In the deterministic limit of our stochastic scheme (tau-leaping), each Markov chain is treated as an occupation number that follows ODEs with the same voltage- and Ca^{2+}-dependent transition rates used in the stochastic model. The macroscopic currents follow by replacing the open-state indicators with their occupation fractions (for I_{CaL}, Equation 14 from main text; and I_{rel}, Equation 15 from main text) and couple to the three-compartment Ca^{2+} system exactly as defined in Equations 3–9 (main text).

2.1 Deterministic equations for the Markov Chain formulation of the L-type calcium channel

The variations of the different states shown in Figure 2 (main text) corresponding to the states in the L-type calcium channel read:

$$\frac{d\boldsymbol{I_f}}{dt} = d_{1-}\boldsymbol{I'_f} + [f_{1-} + f_{ca_1}]\boldsymbol{C} - (d_{1+} + f_{1+})\boldsymbol{I_f} \tag{S3}$$

$$\frac{d\boldsymbol{I'_f}}{dt} = d_{1+}\boldsymbol{I_f} + [f_{1-} + f_{ca_2}]\boldsymbol{C'} + [f_{3-} + f_{ca_3}]\boldsymbol{O} - (d_{1-} + f_{1+} + f_{5+})\boldsymbol{I'_f} \tag{S4}$$

$$\frac{d\boldsymbol{C}}{dt} = f_{1+}\boldsymbol{I_f} + d_{1-}\boldsymbol{C'} + f_{2+}\boldsymbol{I_{f2}} - [f_{1-} + f_{ca_1} + d_{1+} + f_{2-}]\boldsymbol{C} \tag{S5}$$

$$\frac{d\boldsymbol{C'}}{dt} = d_{1+}\boldsymbol{C} + f_{1+}\boldsymbol{I'_f} + d_{3-}\boldsymbol{O} + f_{2+}\boldsymbol{I'_{f2}} - [d_{1-} + f_{1-} + f_{ca_2} + \phi d_{2+} + f_{2-}]\boldsymbol{C'} \tag{S6}$$

$$\frac{d\boldsymbol{I_{f2}}}{dt} = f_{2-}\boldsymbol{C} + d_{1-}\boldsymbol{I'_{f2}} - (f_{2+} + d_{1+})\boldsymbol{I_{f2}} \tag{S7}$$

$$\frac{d\boldsymbol{I'_{f2}}}{dt} = d_{1+}\boldsymbol{I_{f2}} + f_{2-}\boldsymbol{C'} + f_{4-}\boldsymbol{O} - [d_{1-} + f_{2+} + f_{6+}]\boldsymbol{I'_{f2}} \tag{S8}$$

$$\frac{d\boldsymbol{O}}{dt} = f_{5+}\boldsymbol{I'_f} + \phi d_{2+}\boldsymbol{C'} + f_{6+}\boldsymbol{I'_{f2}} - [f_{3-} + f_{ca_3} + d_{3-} + f_{4-}]\boldsymbol{O}. \tag{S9}$$

The MC transition rates are presented in Sections 2.1.1 and 2.1.2.

2.1.1 Equations for $\boldsymbol{d_\bullet}$ rates

$$d_{1+} = d_{inf}/\tau_d \tag{S10}$$

$$d_{1-} = (1 - d_{inf})/\tau_d \tag{S11}$$

$$d_{inf} = \frac{1}{1 + e^{(-8-V)/7.5}} \tag{S12}$$

$$\tau_d = A_d \times B_d + C_d \tag{S13}$$

$$A_d = \frac{1.4}{1 + e^{(-35-V)/13}} + 0.25 \tag{S14}$$

$$B_d = \frac{1.4}{1 + e^{(V+5)/5}} \tag{S15}$$

$$C_d = \frac{1}{1 + e^{(50-V)/20}} \tag{S16}$$

$$d_{2+} = 0.397 \times d_{1+} \quad \text{(S17)} \qquad d_{3-} = 0.793 \times d_{1-} \quad \text{(S18)}$$

where V (mV) is the transmembrane potential.

2.1.2 Equations for $f_\bullet$ rates

$$f_{1+} = f_{inf}/\tau_f \quad \text{(S19)} \qquad f_{1-} = (1 - f_{inf})/\tau_f \quad \text{(S20)}$$

$$f_{inf} = \frac{1}{1 + e^{(V+20)/7}} \quad \text{(S21)} \qquad \tau_f = A_f + B_f + C_f \quad \text{(S22)}$$

$$A_f = 1102.5e^{(-(V+27)^2/225)} \quad \text{(S23)} \qquad B_f = \frac{200}{1 + e^{(13-V)/10}} \quad \text{(S24)} \qquad C_f = \frac{180}{1 + e^{(V+30)/10}} + 20 \quad \text{(S25)}$$

$$f_{2+} = f_{2_{inf}}/\tau_{f_2} \quad \text{(S26)} \qquad f_{2-} = (1 - f_{2_{inf}})/\tau_{f_2} \quad \text{(S27)}$$

$$f_{2_{inf}} = \frac{0.67}{1 + e^{(V+35)/7}} + 0.33 \quad \text{(S28)} \qquad \tau_{f_2} = A_{f_2} + B_{f_2} + C_{f_2} \quad \text{(S29)}$$

$$A_{f_2} = \alpha e^{-(V+\beta)^2/\gamma} \quad \text{(S30)} \qquad B_{f_2} = \frac{31}{1 + e^{(25-V)/10}} \quad \text{(S31)} \qquad C_{f_2} = \frac{\delta}{1 + e^{(V+30)/10}} \quad \text{(S32)}$$

$$f_{3-} = 1.027 \times f_{1-} \quad \text{(S33)} \qquad f_{4-} = 1.279 \times f_{2-} \quad \text{(S34)}$$

$$f_{5+} = 0.415 \times f_{1+} \quad \text{(S35)} \qquad f_{6+} = 0.739 \times f_{2+} \quad \text{(S36)}$$

$$f_{ca_1} = 1.162 \times f_{ca}(c) \quad \text{(S37)} \qquad f_{ca_2} = 1.620 \times f_{ca}(c) \quad \text{(S38)} \qquad f_{ca_3} = 1.369 \times f_{ca}(c) \quad \text{(S39)}$$

$$f_{ca}(c) = \frac{1}{1 + (1.293 \times \bar{c_p}/c)^{(3\times 0.688)}} \quad \text{(S40)}$$

where V(mV) is transmembrane potential; c (mM) is $[Ca]_{ss}$; and $\bar{c_p} = 3.0$ (mM) is a threshold for calcium dependence.

2.2 Deterministic equations for the Markov chain formulations of the RyR states occupation

The variations of the different states shown in Figure 3 (main text) corresponding to the states in the RyR channel read:

$$\frac{d\boldsymbol{I}}{dt} = k_1[Ca]_{ss}^2\boldsymbol{RI} + k_2[Ca]_{ss}\boldsymbol{O} - (k_3 + k_4)\boldsymbol{I}, \tag{S41}$$

$$\frac{d\boldsymbol{R}}{dt} = k_3\boldsymbol{O} + k_4\boldsymbol{RI} - (k_1[Ca]_{ss}^2 + k_2[Ca]_{ss})\boldsymbol{R}, \tag{S42}$$

$$\frac{d\boldsymbol{RI}}{dt} = k_2[Ca]_{ss}\boldsymbol{R} + k_3\boldsymbol{I} - (k_1[Ca]_{ss}^2 + k_4)\boldsymbol{RI}, \tag{S43}$$

$$\frac{d\boldsymbol{O}}{dt} = k_1[Ca]_{ss}^2\boldsymbol{R} + k_4\boldsymbol{I} - (k_2[Ca]_{ss} + k_3)\boldsymbol{O}; \tag{S44}$$

3 COMPUTATIONAL COST: EXECUTION TIME ANALYSIS

In this section, we present the execution times required for a typical simulation run in which calcium release statistics are computed. Execution times are shown for representative cases corresponding to a small, intermediate, and large number of L-type calcium channels ($N_{\text{LCC}} = 4, 256, 16384$), as well as for the deterministic formulation of the model.

All timings correspond to simulations of 100 pacing cycles and are reported as wall-clock execution time.

Table S1. Execution time required to simulate 100 pacing cycles for representative stochastic and deterministic configurations.

Model configuration	Execution time (s)
Stochastic SA-CaRU, $N_{\text{LCC}} = 4$	1091.56
Stochastic SA-CaRU, $N_{\text{LCC}} = 256$	1170.02
Stochastic SA-CaRU, $N_{\text{LCC}} = 16384$	1286.21
Deterministic model	544.82

All simulations were performed on a Linux computer running Ubuntu 24.04.3 LTS (x86_64), equipped with an Intel Core i7–3630QM processor (4 physical cores, 8 threads, base frequency 2.40 GHz, turbo up to 3.40 GHz) and 8 GB of RAM. The code was compiled using GCC 13.3.0 with standard optimization flags and executed in single-threaded mode. Execution times reported correspond to wall-clock time measured on this system.
